# Effects of Aerobic Exercise on Endogenous Estrogens and Body Composition in Women with Overweight or Obesity: A Systematic Review and Meta-Analysis

**DOI:** 10.3390/metabo16100725

**Published:** 2026-09-28

**Authors:** Chao Xie, Mingnan Zhuang, Jiansong Huang, Yiran Guo

**Affiliations:** 1 School of Sports Training, Tianjin University of Sport, Tianjin 301617, China; 2 Department of Winter Sports, Hebei Sport University, Shijiazhuang 050041, China; 3 Department of Hematology, The First Affiliated Hospital, Zhejiang University School of Medicine, Hangzhou 311121, China

**Keywords:** aerobic exercise, overweight and obesity, endogenous estrogens, body composition, meta-analysis

## Abstract

**Purpose:** Overweight and obesity may alter endogenous estrogen exposure in women, yet evidence on the effects of aerobic exercise alone remains limited. This systematic review and meta-analysis aimed to evaluate the effects of aerobic exercise on endogenous estrogen profiles and body composition in adult women with overweight or obesity. **Methods:** We systematically searched PubMed, Web of Science, Scopus, Embase, and the Cochrane Library for randomized controlled trials involving women aged ≥ 18 years with BMI ≥ 25 kg/m^2^ who received aerobic exercise or aerobic exercise-based interventions. Random-effects meta-analyses used the DerSimonian–Laird estimator with Hartung–Knapp adjustment; subgroup analyses, meta-regression, sensitivity analyses, publication-bias assessment, and GRADE were also performed. **Results:** Fifteen trials involving 1217 participants were included. Aerobic exercise was associated with lower E_2_ (Hedges’ g = −0.22, 95% CI −0.38 to −0.06, *p* < 0.05), E_1_ (g = −0.19, 95% CI −0.33 to −0.04, *p* < 0.05), and free E_2_ (g = −0.18, 95% CI −0.33 to −0.04, *p* < 0.05), and with higher SHBG (g = 0.49, 95% CI 0.06 to 0.92, *p* < 0.05). Aerobic exercise was also associated with reductions in body weight (MD = −3.06, 95% CI −4.60 to −1.51, *p* < 0.05), BMI (MD = −1.48, 95% CI −2.91 to −0.06, *p* < 0.05), waist circumference (MD = −2.50, 95% CI −3.59 to −1.41, *p* < 0.05), and total fat mass (MD = −2.30, 95% CI −3.48 to −1.12, *p* < 0.05). Heterogeneity was substantial for several outcomes, and the certainty of evidence ranged from moderate to very low. **Conclusions:** These findings suggest that aerobic exercise may be associated with changes in estrogen bioavailability and reductions in body composition measures, but the evidence remains preliminary.

## 1. Introduction

Overweight and obesity are no longer regarded merely as conditions of increased body weight; rather, they are recognized as complex chronic diseases characterized by excessive adipose tissue accumulation, metabolic dysfunction, and chronic low-grade inflammation [1]. Since the beginning of the 21st century, overweight and obesity have become major global public health concerns. Obesity is closely associated with chronic diseases, including type 2 diabetes, cardiovascular disease, and several malignancies [2,3]. In women, obesity-related adipose tissue expansion and metabolic disturbances may also disrupt sex hormone homeostasis [4,5], particularly by altering endogenous estrogen levels and bioavailability. Endogenous estrogens, as key components of the female sex hormone milieu [6,7,8], regulate the menstrual cycle and follicular development through the hypothalamic–pituitary–gonadal axis and influence insulin sensitivity and inflammatory responses via central and peripheral pathways [9,10]. Therefore, in women with overweight or obesity, estrogen-related markers may reflect not only reproductive endocrine status but also endocrine links among adipose tissue function, metabolic health, and the risk of obesity-related diseases.

Adipose tissue is both an important target organ for estrogen action and a major site of peripheral estrogen production. Adipose tissue expansion may enhance aromatase activity, thereby promoting the conversion of androgens into estrogens [11]. In parallel, obesity-related insulin resistance and chronic low-grade inflammation may reduce sex-hormone-binding globulin (SHBG) levels, increasing the proportion of free estrogen and altering estrogen bioavailability [12,13]. These mechanisms suggest that obesity may affect not only total circulating estrogen concentrations but also estrogen production, binding status, and tissue availability. This relationship may be particularly relevant in postmenopausal women, in whom ovarian estrogen secretion declines markedly and adipose-derived estrogen becomes an increasingly important source of circulating estrogens [5]. Accordingly, in individuals with high body mass, adipose tissue expansion may increase adipose-derived estrogen production while also altering estrogen receptor expression and tissue sensitivity to estrogen signaling. The dysregulation of estrogen production and signaling may therefore contribute to obesity-related metabolic and hormone-associated diseases [14,15]. Based on this mechanistic framework, relying solely on individual total estrogen markers, such as E_1_ or E_2_, may be insufficient to fully characterize estrogen exposure in women with obesity. Instead, evaluating E_1_, E_2_, SHBG, free estradiol, and body composition within an integrated evidence framework may provide a more comprehensive understanding of obesity-related changes in estrogen bioavailability.

Current interventions for obesity-related endocrine and metabolic abnormalities primarily include pharmacological therapies and lifestyle interventions [16,17,18]. However, pharmacological approaches may be limited by age, population characteristics, symptom severity, safety concerns, and scalability [19,20]. By contrast, exercise, as a core component of lifestyle intervention, may improve body composition and adipose tissue function, thereby contributing to the regulation of obesity-related endogenous estrogen levels [21,22]. Exercise also improves cardiorespiratory fitness, enhances skeletal muscle insulin sensitivity, and modulates the endocrine function of adipose tissue [23,24], suggesting that it may influence estrogen production, sex-hormone-binding globulin (SHBG) levels, and estrogen bioavailability through mechanisms that are not solely dependent on weight loss [25]. Among different exercise modalities, aerobic exercise is of particular interest. Characterized by sustained, rhythmic activity involving large muscle groups, aerobic exercise effectively increases energy expenditure and promotes fat mobilization and oxidation. It is therefore widely used, scalable, and generally well tolerated for weight management and metabolic improvement in individuals with overweight or obesity [26,27]. Accordingly, aerobic exercise may serve not only as a weight management strategy but also as a non-pharmacological approach to modulating estrogen exposure and bioavailability in women with obesity, potentially through improvements in body composition and SHBG-related hormone-binding status.

Despite the established role of aerobic exercise in obesity management, its effects on the endogenous estrogen environment in women with overweight or obesity remain incompletely understood. Several limitations exist in the current literature. First, study populations have often been insufficiently specific. Previous studies have primarily focused on generally healthy women or mixed female cohorts [28,29]. However, women with overweight or obesity should not be regarded simply as a high-body-mass population. Their adipose tissue expansion, insulin resistance, and chronic inflammatory state may confer distinct patterns of estrogen production, binding, and bioavailability compared with women of normal weight [30]. Moreover, obesity-related endocrine alterations are unlikely to be uniform across all women; menopausal status, underlying metabolic conditions, and clinical phenotypes may further influence estrogen levels and responses to exercise interventions. Women across different menopausal stages were therefore considered relevant to this review because the sources and regulation of endogenous estrogen differ before and after menopause, while overweight and obesity may influence estrogen production and bioavailability across these stages. Rather than restricting the review to a single menopausal group, menopausal status was examined as a potential source of variation in intervention effects. Second, the independent effects of exercise itself require further clarification. Many previous studies have used comprehensive lifestyle programs combining dietary control and exercise. Although such interventions help characterize the overall effects of weight management on endogenous estrogen levels, their results may be confounded by dietary restriction, changes in energy intake, and weight loss. Therefore, it is necessary to isolate the specific effects of aerobic exercise on estrogen levels and binding proteins [31]. Third, the existing evidence remains fragmented. Previous research has mainly reported changes in individual endogenous estrogen markers, such as total estradiol and estrone, with less attention to the relationships among sex-hormone-binding globulin (SHBG), free estradiol, and body composition changes [22]. SHBG regulates estrogen bioavailability, whereas adipose tissue, a key site of estrogen production, metabolism, and action, is itself regulated by estrogen signaling [32]. Consequently, focusing solely on total estrogen levels may not fully capture how exercise interventions affect estrogen bioavailability and related metabolic characteristics. Accordingly, the novelty of the present review lies in its specific focus on women with overweight or obesity, its emphasis on randomized controlled trials in which the independent effect of aerobic exercise could be isolated, and its integrated evaluation of endogenous estrogen markers, SHBG, free estradiol, and body composition-related outcomes.

Given these limitations, this systematic review and meta-analysis aimed to synthesize evidence from randomized controlled trials (RCTs) evaluating the effects of aerobic exercise on endogenous estrogen profiles and body composition in adult women with overweight or obesity. The primary research question was whether aerobic exercise, compared with eligible control conditions, is associated with changes in endogenous estrogen-related markers, including estradiol (E_2_), estrone (E_1_), sex-hormone-binding globulin (SHBG), and free estradiol (Free E_2_), in this population. Particular attention was given to changes in E_2_ and E_1_, while SHBG and Free E_2_ were incorporated to provide additional information on estrogen-binding status and bioavailability. Body composition was considered both as an outcome of aerobic exercise and, in exploratory analyses, as a potential explanatory factor associated with variation in estrogen responses; it was not assumed to represent a confirmed mediator or causal mechanism. In addition, subgroup analyses and exploratory meta-regression were conducted to investigate whether menopausal status, baseline health conditions, exercise prescription parameters, and changes in body composition contributed to variations in effects across studies. Overall, this review sought to clarify whether aerobic exercise is associated with changes in endogenous estrogen exposure and bioavailability in women with overweight or obesity and to provide a more integrated evidence base for future mechanistic and clinical research.

## 2. Materials and Methods

This systematic review and meta-analysis was registered with PROSPERO (registration number: CRD420261365227) and was conducted in accordance with the Preferred Reporting Items for Systematic Reviews and Meta-Analyses (PRISMA 2020) guidelines [33].

### 2.1. Search Strategy

Before the formal literature search, one researcher (C.X.) conducted a preliminary database screening. Subsequently, two independent researchers (C.X. and M.N.Z.) performed a comprehensive search of five major databases—Web of Science, PubMed, Scopus, Embase, and the Cochrane Library—on 27 March 2026. To maximize the comprehensiveness of study identification, three snowballing strategies were also applied: (1) screening the reference lists of included studies; (2) identifying subsequent studies that cited the included articles; and (3) using the “similar articles” functions in PubMed and Embase to identify related literature. The detailed search strings are provided in Supplementary Material SB.

### 2.2. Study Screening and Inclusion Criteria

Initially, one researcher (C.X.) used EndNote X9 to conduct preliminary screening and remove duplicate records. After deduplication, two independent researchers (C.X. and M.N.Z.) used Zotero 7.0.22 to screen articles according to predefined inclusion and exclusion criteria. Disagreements during screening were resolved through discussion; if consensus could not be reached, a third researcher (J.H.) adjudicated and made the final decision. The inclusion criteria were based on the PICOS framework: population, women with overweight or obesity; intervention, aerobic exercise; comparator, usual daily activities or other eligible control conditions; outcomes, endogenous estrogen-related markers; and study design, randomized controlled trials. The specific criteria are presented in Table 1.

### 2.3. Data Extraction and Conversion

Two researchers (C.X. and M.N.Z.) independently extracted data from studies that met the inclusion criteria using a predesigned Microsoft Excel spreadsheet. The extracted information included: (1) basic study characteristics, including first author, study region, and publication year; (2) baseline participant characteristics, including age, body mass index (BMI), menopausal status, and health status; and (3) intervention characteristics, including exercise modality, intervention duration, exercise intensity, intervention frequency, single-session duration, and weekly exercise volume. Because exercise intensity was reported using different metrics across the included studies, such as 45–65% of maximum heart rate (HRmax), 50–80% of heart rate reserve (HRR), and 50–70% of maximal oxygen uptake (VO_2_max), intensities were standardized according to the American College of Sports Medicine (ACSM) guidelines [34] and categorized as low, moderate, or high to facilitate subsequent statistical analyses. In addition, to evaluate cumulative weekly exposure to exercise, weekly exercise volume was calculated from exercise frequency (sessions/week) and single-session duration (min/session) when it was not directly reported in the original study [35].

Weekly exercise volume (min/week) = Exercise frequency (sessions/week) × Duration per session (min/session)

When data were presented only in graphical form, the corresponding authors were first contacted to request the raw data. If no response was received and the relevant data could be extracted from the available figures, WebPlotDigitizer 4.1 was used for data extraction. Studies for which complete data could not be obtained were excluded from the analysis.

In various studies, if only the following statistics are provided, they are converted into the standard deviation using the formulas below:

① Conversion from the confidence interval (CI) [36]:

$$\mathrm{SD}=\sqrt{N}\frac{{\mathrm{CI}}_{\mathrm{high}}-{\mathrm{CI}}_{\mathrm{low}}}{2t}$$

where SD represents the standard deviation, N represents the sample size, and CI_high_ and CI_low_ represent the upper and lower limits of the reported confidence interval, respectively. The term *t* represents the critical value of Student’s *t*-distribution corresponding to the confidence level reported in the original study, with degrees of freedom calculated as *df* = *N* − 1. For a 95% confidence interval, t corresponds to t_0.975,N−1._

② Calculated from the standard error (SE) [36]:

$$\mathrm{SD}=\sqrt{N}\times \mathrm{SE}$$

For the difference between pre- and post-intervention mean values for the same group of subjects in a research report:

*MD_diff_* = *M_post_* − *M_pre_*

Here, the terms represent the raw mean difference, the reported post-intervention mean, and the reported pre-intervention mean, respectively.

If the study does not directly report the standard deviation (SD) of the pre–post difference, it is estimated using the following formula:

$${\mathrm{SD}}_{\mathrm{diff}}=\sqrt{{{\mathrm{SD}}_{\mathrm{pre}}}^{2}+{{\mathrm{SD}}_{\mathrm{post}}}^{2}-2r\times {\mathrm{SD}}_{\mathrm{pre}}\times {\mathrm{SD}}_{\mathrm{post}}}$$

For studies that did not directly report the standard deviation (SD) of change scores from pre- to post-intervention, the SD of the change was estimated using the baseline and follow-up SDs together with an assumed pre–post correlation coefficient (r), which represents the correlation between repeated measurements obtained from the same participants before and after the intervention. Because most original studies did not provide sufficient information to estimate this coefficient directly, r = 0.5 was used in the primary analysis. Given that the true pre–post correlation may vary across outcomes, sensitivity analyses were performed using alternative correlation coefficients of r = 0.2 and r = 0.8 for the main outcomes in which the change-score SD required imputation. The pooled effect estimates, 95% confidence intervals (CIs), and statistical significance obtained under these alternative assumptions were compared with those from the primary analysis (r = 0.5) to evaluate the sensitivity of the findings to the assumed pre–post correlation [37].

### 2.4. Outcome Measures

Estradiol (E_2_) and estrone (E_1_) were defined as the primary outcome measures. To provide a more comprehensive assessment of changes in endogenous estrogen profiles in women with overweight or obesity, sex-hormone-binding globulin (SHBG), free estradiol (Free E_2_), and body composition-related indicators were included as secondary outcomes. Body composition-related indicators included body weight, body mass index (BMI), waist circumference (WC), and total fat mass (TFM).

### 2.5. Risk-of-Bias Assessment

Two researchers independently assessed the included studies using the Cochrane Risk of Bias 2 (RoB 2) tool. The RoB 2 tool evaluates bias arising from the randomization process, deviations from intended interventions, missing outcome data, outcome measurement, and selection of the reported results. Each study was classified as having a low risk of bias, some concerns, or a high risk of bias based on domain-level assessments. Disagreements were resolved through discussion or, when necessary, consultation with a third researcher.

### 2.6. Assessment of Evidence Quality

Two researchers independently assessed the certainty of evidence using the Grading of Recommendations Assessment, Development and Evaluation (GRADE) approach. The certainty of evidence was classified as high, moderate, low, or very low. Because all included studies were randomized controlled trials, the initial certainty of evidence was rated as high. The assessment considered five domains: (1) Risk of bias was downgraded by one level if the overall risk of bias for a given outcome was judged as having some concerns and by two levels if judged as high. (2) Inconsistency was evaluated by considering the magnitude of statistical heterogeneity (I^2^), the direction and magnitude of study-specific effects, the overlap of confidence intervals, and whether heterogeneity could be explained by prespecified subgroup analyses. Certainty was downgraded by one level when substantial heterogeneity, generally corresponding to I^2^ ≥ 50%, remained unexplained after consideration of these factors. A two-level downgrade was reserved for very serious inconsistency, generally characterized by considerable heterogeneity (I^2^ ≥ 75%) together with marked and unexplained variation in the magnitude or direction of effects. (3) Indirectness was downgraded by one level when the study population, intervention, comparator, or outcome partially deviated from the PICO criteria of this review in a way that could affect the applicability of the findings. (4) Imprecision was assessed by considering the width of the 95% confidence interval, the range of effect sizes compatible with the interval, and the amount of information contributing to the estimate. Certainty was downgraded when the confidence interval was sufficiently wide to include materially different interpretations of the effect, particularly when the estimate was based on a limited number of studies or participants. Statistical significance alone was not used as a criterion for downgrading. (5) Publication bias was downgraded by one level when Egger’s test suggested potential publication bias (*p* < 0.05).

### 2.7. Statistical Methods

Statistical analyses and figure generation were performed using R software (version 4.5.2). Because some studies reported multiple effect sizes, we first compared the fit of conventional two-level random-effects models with that of multilevel random-effects models. Model performance was evaluated using the Akaike information criterion (AIC), Bayesian information criterion (BIC), and likelihood-ratio tests [38]. For studies with multiple intervention groups sharing a single control group, data were handled according to the specific analytical objective. When multiple intervention groups belonged to the same intervention category and could be combined, they were pooled. When distinct intervention groups needed to be retained for separate comparisons, the shared control group sample size was divided equally across comparisons, in accordance with the recommendations of the *Cochrane Handbook*, to avoid double counting. If the multilevel model did not significantly improve model fit, a conventional two-level random-effects model was used as the primary analytical model. Conversely, when the multilevel model provided a substantially better fit, it was used to account for correlations among multiple effect sizes within the same study.

Effect estimates were pooled using inverse-variance weighting within a random-effects framework. Between-study variance (τ^2^) was estimated using the DerSimonian–Laird method, and the Hartung–Knapp method was applied to adjust the confidence intervals and significance tests for pooled effect estimates [39,40]. Given the relatively small number of studies included for several outcomes, the Hartung–Knapp adjustment was used to provide more conservative inference than conventional Wald-type confidence intervals. Heterogeneity was assessed using Cochran’s Q test, I^2^, and τ^2^, and 95% confidence intervals for heterogeneity estimates were calculated using the Jackson method. Appropriate effect-size metrics were selected according to outcome characteristics. For continuous hormone-related outcomes measured using different units or scales across studies, the standardized mean difference (SMD) was used [41]. For body composition outcomes measured on consistent scales across studies, such as body weight and body mass index (BMI), the mean difference (MD) was used [42]. To account for small sample sizes in some studies, Hedges’ g was applied as a small-sample bias correction for SMDs. Standardized effect sizes were interpreted according to Cohen’s criteria: values of approximately 0.2 indicated a negligible effect, 0.2–0.5 a small effect, and 0.5–0.8 a moderate effect. MDs were interpreted according to their original units and clinical relevance.

For interpretation of between-study heterogeneity, I^2^ was considered together with τ^2^, the direction and magnitude of study-specific effects, and the overlap of confidence intervals. Prediction intervals were also calculated to illustrate the expected range of effects in future studies [43]. I^2^ values of approximately 0–40% were considered potentially unimportant, 30–60% may represent moderate heterogeneity, 50–90% may represent substantial heterogeneity, and 75–100% may represent considerable heterogeneity. Because these ranges overlap, I^2^ values were not interpreted using rigid thresholds alone [44].

To further explore potential sources of heterogeneity, subgroup and meta-regression analyses were conducted according to methodological recommendations by Ruppar et al. [45]. Subgroup analyses were performed only when at least five studies were available, whereas meta-regression analyses were conducted only when at least ten studies were available and were considered exploratory, given the relatively limited number of contributing studies/effect sizes and the examination of multiple potential moderators. These analyses were not performed when the number of studies for a given outcome was insufficient. Different analytical strategies were applied according to variable type. Subgroup analyses were used for categorical variables, including intervention type, menopausal status, health status, detection method, fasting status, and exercise intensity. For health-status subgroup analyses, otherwise healthy women with overweight or obesity were distinguished from women with specific clinical conditions, including PCOS, breast cancer, functional dyspepsia, and infertility, where sufficient data were available. Meta-regression analyses were used for continuous variables, including BMI, age, intervention duration, exercise frequency, single-session duration, weekly exercise volume, and changes in BMI and body weight [46]. Meta-regression analyses were performed within a mixed-effects model framework, with model parameters estimated using restricted maximum likelihood (REML). Compared with conventional maximum likelihood estimation, REML reduces bias in the estimation of random-effects variance components by accounting for the uncertainty associated with fixed-effect estimates and is therefore generally considered more robust [47]. The results of subgroup and meta-regression analyses were also considered when evaluating whether the observed between-study heterogeneity could be reasonably explained.

To examine the robustness of the pooled results and identify studies with a potentially disproportionate influence on the overall findings, influence diagnostics were first performed. Cook’s distance was used to assess the influence of individual studies on pooled effect estimates and model parameters. A Cook’s distance value substantially higher than those of other studies was considered to indicate a potentially influential study. Leave-one-out sensitivity analyses were then conducted by sequentially excluding one study at a time and re-running the meta-analysis to evaluate changes in pooled effect estimates and heterogeneity indices. Publication bias was assessed using funnel plots and Egger’s test [48,49]. Statistical tests for publication bias were not performed when the number of studies for a specific outcome was insufficient; in such cases, publication bias was assessed descriptively or not evaluated. A *p* value > 0.05 was interpreted as indicating no statistically significant evidence of publication bias, whereas *p* < 0.05 was considered statistically significant. *p* values between 0.05 and 0.10 were interpreted as indicating a trend toward statistical significance.

## 3. Results

### 3.1. Literature Search Results

**Figure 1:**
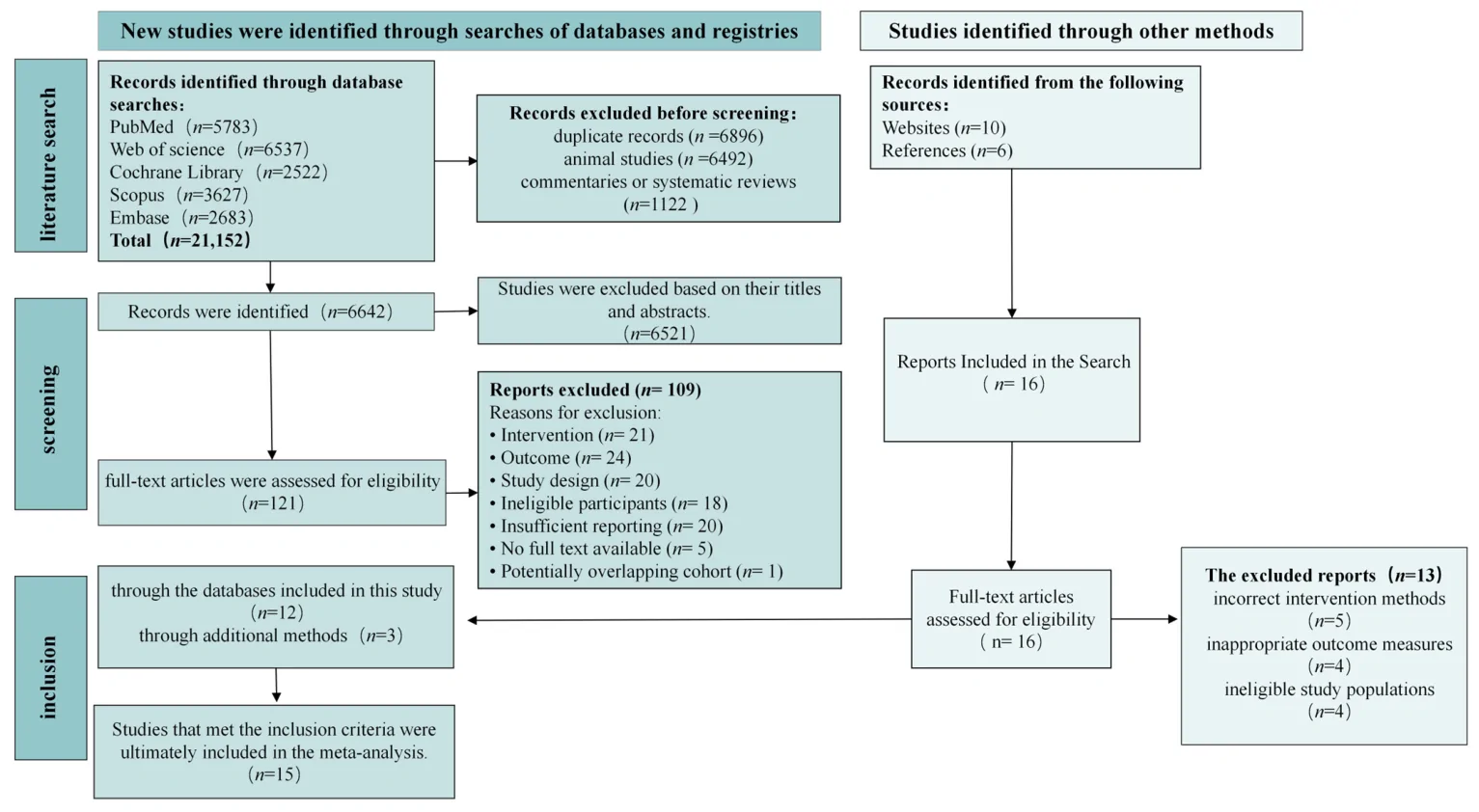
PRISMA flow diagram for included and excluded study.

The initial database search identified 21,152 records from PubMed, Web of Science, the Cochrane Library, Scopus, and Embase. After duplicate removal and title/abstract screening, 121 reports underwent full-text assessment. Of these, 109 were excluded, resulting in 12 studies being included through the database search pathway. One report was excluded because of potential cohort overlap with an included study, as indicated by identical analyzed group sizes and identical baseline means and standard deviations for age, height, weight, BMI, and HOMA-IR in both groups. Although the reports listed different trial registration numbers, sample independence could not be adequately established from the available information; the report was therefore conservatively excluded to avoid potential double counting of participants. In addition, 16 records were identified through secondary searches, of which 13 were excluded and 3 additional studies were included. Therefore, a total of 15 randomized controlled trials met the eligibility criteria and were included in the review [50,51,52,53,54,55,56,57,58,59,60,61,62,63,64]. The study selection process and screening results are shown in Figure 1.

### 3.2. Characteristics of Included Studies

All 15 included studies were randomized controlled trials [50,51,52,53,54,55,56,57,58,59,60,61,62,63,64], comprising a total of 1217 participants. Individual group sizes ranged from 8 to 160 participants, whereas study-level sample sizes ranged from 18 to 319 participants across the groups included in this review, with shared control groups counted only once. Participant age ranged from 21.7 to 71.7 years, and body mass index (BMI) ranged from 25.0 to 37.88 kg/m^2^. All participants were women with overweight or obesity, although they varied in menopausal status and baseline health conditions. Regarding menopausal status, 245 participants were premenopausal women (20.13%), 882 were postmenopausal women (72.47%), and 60 were peri-menopausal women (4.93%); the remaining 30 participants (2.47%) were enrolled in a study with a mixed menopausal cohort. Regarding baseline health status, most participants were otherwise healthy women with overweight or obesity (*n* = 910, 74.77%), whereas some studies included women with specific diseases or clinical conditions, including polycystic ovary syndrome (*n* = 199, 16.35%), breast cancer (*n* = 30, 2.47%), functional dyspepsia (*n* = 60, 4.93%), and infertility (*n* = 18, 1.48%). The basic characteristics of the included studies are presented in Table 2.

All 15 included studies used aerobic exercise or aerobic-dominant interventions. Because some studies included multiple exercise intervention groups, 16 aerobic exercise protocols were analyzed for their effects on endogenous estrogen levels in women with overweight or obesity. The exercise modalities included jogging (six protocols) [50,51,55,59,64], combined aerobic exercise (five protocols) [52,53,58,60,61], cycling (two protocols) [54,62], and walking (three protocols) [56,57,63]. Intervention frequency generally ranged from three to six sessions per week, and session duration typically ranged from 25 to 60 min. Intervention duration ranged from 2 to 52 weeks, with 12 weeks being the most common duration. A total weekly exercise volume of 225 min was the most frequently reported dose (Table 3).

### 3.3. Risk-of-Bias Assessment of Included Studies

**Figure 2:**
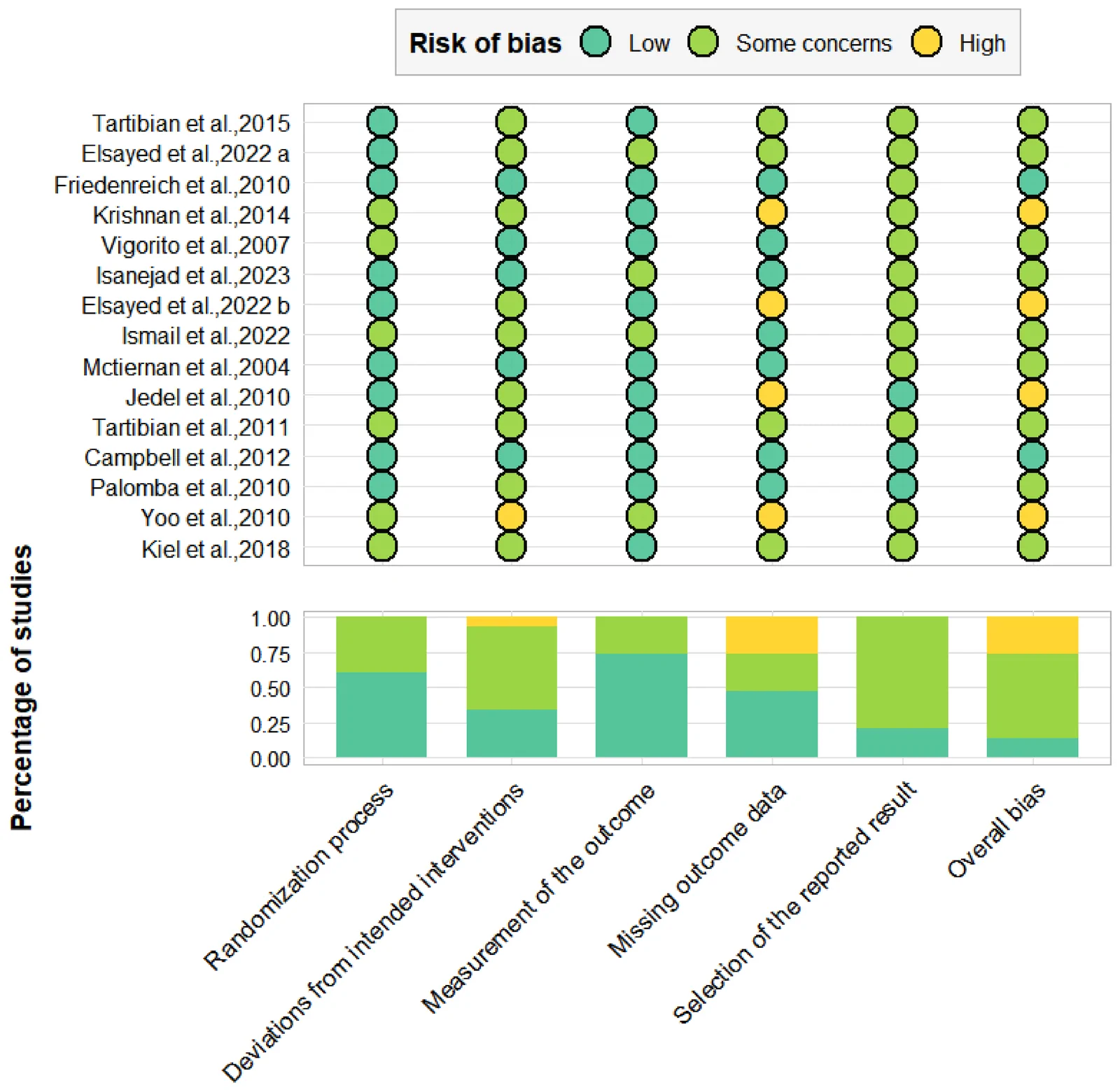
Risk of bias assessment of included studies [50,51,52,53,54,55,56,57,58,59,60,61,62,63,64].

The overall risk of bias across the included studies showed some concerns, with four of the 15 included studies (26.7%) judged to have an overall high risk of bias and two studies (13.3%) judged to have a low risk of bias. The high risk of bias was primarily driven by concerns related to missing outcome data and deviations from intended interventions. Across specific RoB 2 domains, the risk of bias in outcome measurement was generally low, likely because outcomes such as estrogen levels, sex-hormone-binding globulin (SHBG), and body composition were assessed using objective measurement methods. In contrast, some concerns were identified in the domains of deviations from intended interventions and missing outcome data, mainly due to the inherent challenges of implementing exercise interventions and incomplete reporting related to adherence, attrition, or outcome assessment. For the randomization process and selection of the reported results, most studies were rated as having a low risk of bias or some concerns. Detailed results are presented in Figure 2.

### 3.4. Meta-Analysis Results

**Figure 3:**
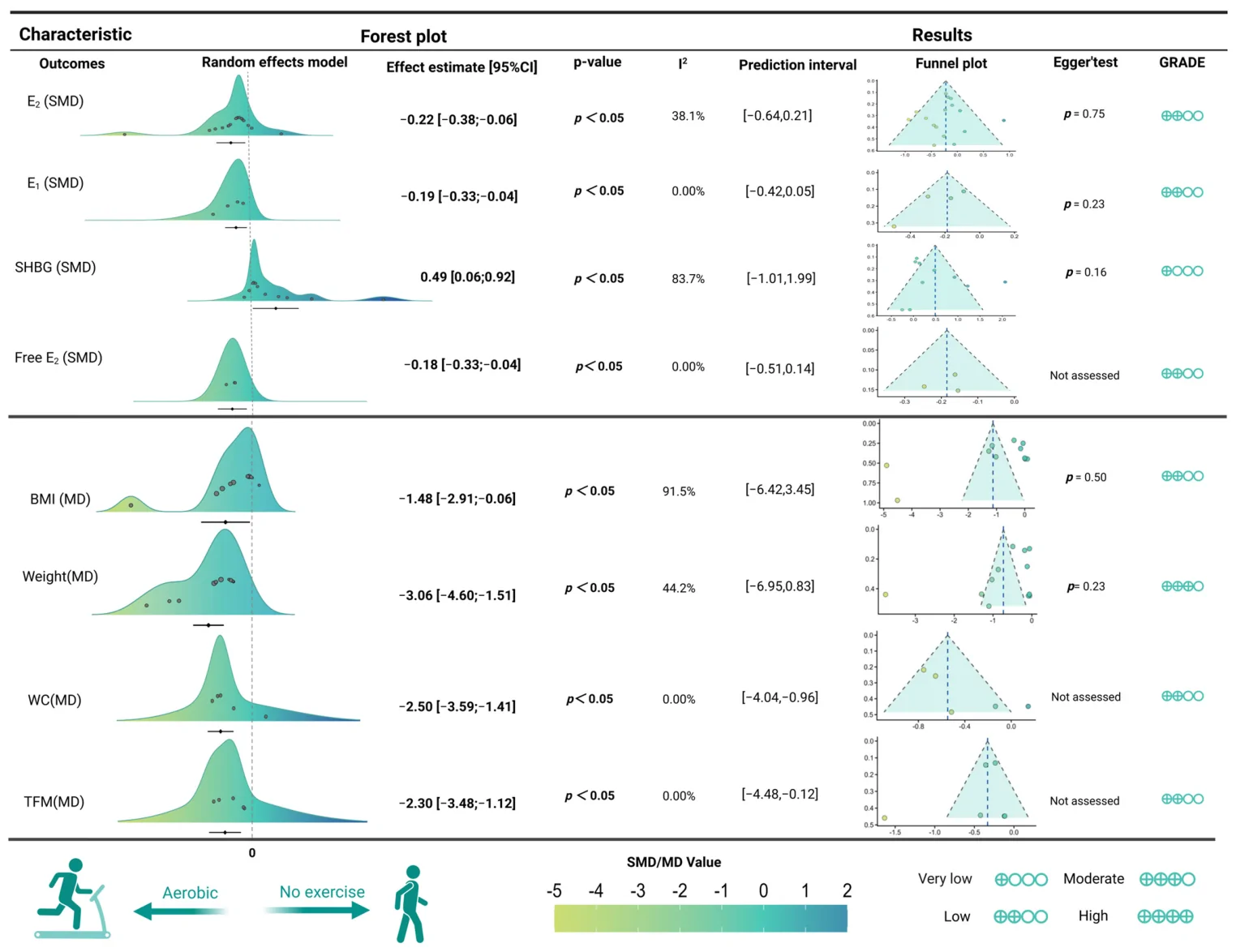
Evidence summary of the effects of aerobic exercise on endogenous estrogens, SHBG, free estradiol, and body composition outcomes. Figure note: The direction of effect differs according to the outcome. For E_2_, E_1_, free E_2_, BMI, body weight, WC, and TFM, negative values indicate lower levels in the aerobic exercise group compared with the no-exercise/control group, suggesting a reduction after aerobic exercise. For SHBG, positive values indicate higher levels in the aerobic exercise group compared with the no-exercise/control group, suggesting an increase after aerobic exercise. Hormonal outcomes are expressed as standardized mean differences (SMDs), whereas body composition outcomes are expressed as mean differences (MDs); in both panels, the dashed vertical line at 0 represents the null effect. SMD, standardized mean difference; MD, mean difference; CI, confidence interval; E_2_, estradiol; E_1_, estrone; SHBG, sex-hormone-binding globulin; WC, waist circumference; TFM, total fat mass.

Before pooling the main effects, we compared the fit of the conventional two-level random-effects model with that of the multilevel random-effects model. The conventional two-level model produced lower Akaike information criterion (AIC) and Bayesian information criterion (BIC) values. In addition, the likelihood-ratio test did not indicate a significant improvement in model fit for the multilevel model compared with the conventional two-level model. Therefore, the conventional two-level random-effects model was used for the primary analysis(see Figure 3). Detailed results are provided in Supplementary Material SC.

#### 3.4.1. Effects of Aerobic Exercise on Endogenous Estrogens

Estradiol (E_2_) was analyzed as a primary marker of endogenous estrogens. A total of 15 studies, comprising 16 effect sizes and 1217 participants, were included in the analysis. Meta-analysis using a DerSimonian–Laird random-effects model with Hartung–Knapp-adjusted confidence intervals showed a significant reduction in E_2_ levels following aerobic exercise interventions (Hedges’ g = −0.22; 95% CI: −0.38 to −0.06; 95% PI: −0.64 to 0.21; *p* < 0.05). However, moderate between-study heterogeneity was observed (I^2^ = 38.1%), indicating moderate variability in effect estimates. In addition, the prediction interval crossed the null value (95% PI: −0.64 to 0.21), suggesting that the magnitude and direction of effects may vary across future study populations and clinical settings. Therefore, this finding should be interpreted with caution.

Subgroup analysis showed that differences between the examined subgroups did not reach statistical significance. In the health-status subgroup analysis, otherwise healthy women with overweight or obesity were analyzed separately from women with specific clinical conditions, including PCOS, breast cancer, functional dyspepsia, and infertility. No statistically significant difference was observed among these health-status subgroups (*p* for subgroup difference = 0.7103; Supplementary Material SE). Similarly, subgroup differences according to detection method, menopausal status, fasting status, exercise intensity, and exercise type were not statistically significant (*p* > 0.05). However, because several clinical subgroups were represented by a limited number of studies, the absence of a statistically significant subgroup difference should be interpreted cautiously and should not be considered evidence that these populations are clinically or biologically equivalent.

**Figure 4:**
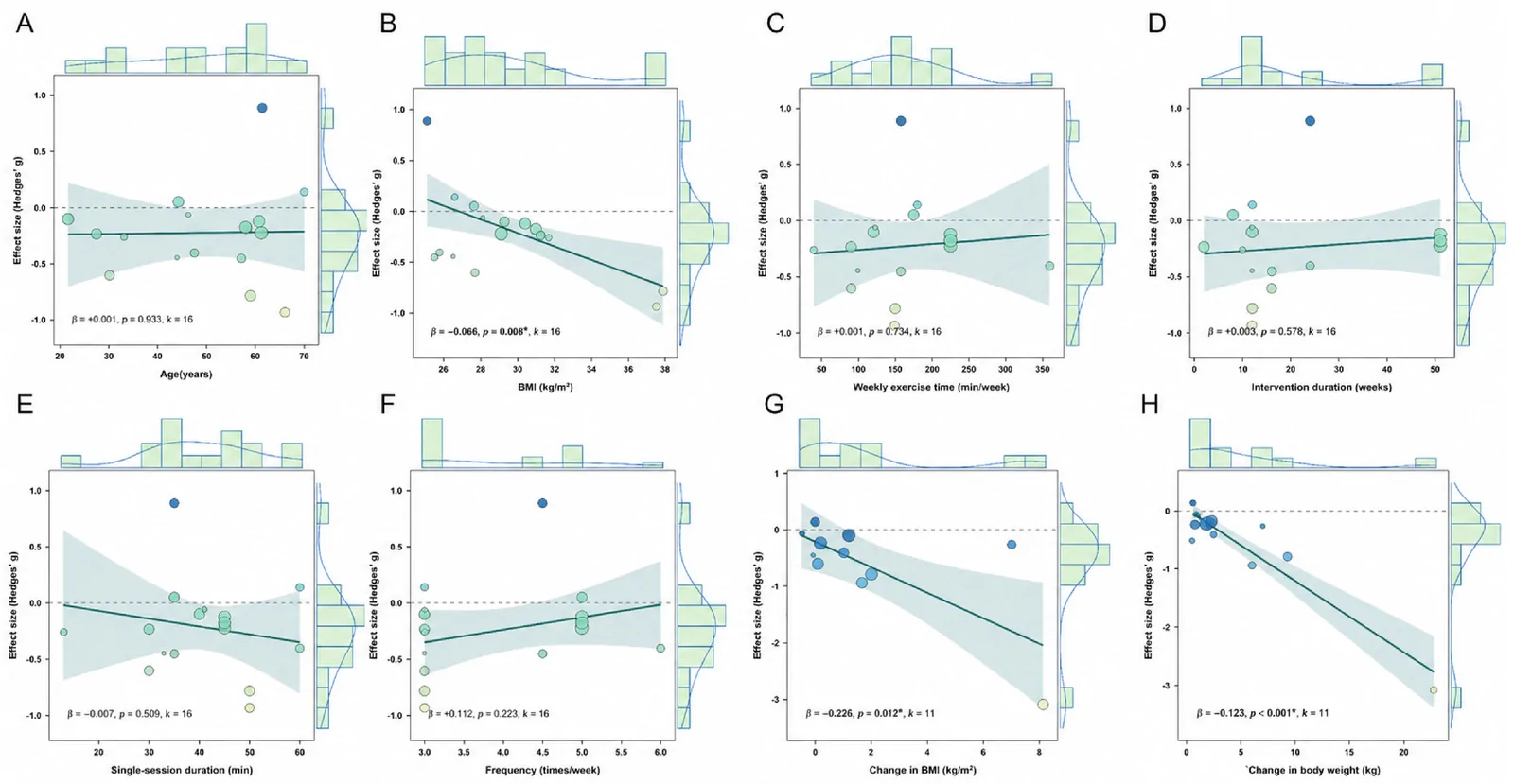
Meta-regression summary plot: effects of aerobic exercise on estradiol (E_2_). Figure note: Panels (**A**–**H**) show meta-regression analyses examining the associations between Hedges’ g and the following moderators: age (years), BMI (kg/m^2^), weekly exercise time (min/week), intervention duration (weeks), single-session duration (min), intervention frequency (times/week), change in BMI (kg/m^2^), and change in body weight (kg), respectively. Each bubble represents an individual effect size, with bubble size proportional to study weight. The solid line indicates the fitted meta-regression line, and the shaded area represents the 95% confidence interval. Marginal histograms and density curves display the distributions of the moderators and effect sizes. β represents the regression coefficient, p represents the significance level, and k represents the number of included effect sizes. An asterisk (*) indicates statistical significance (*p* < 0.05).

Exploratory meta-regression suggested nominal associations of baseline BMI, changes in BMI, and changes in body weight with changes in E_2_ levels (*p* < 0.05), whereas age, exercise frequency, intervention duration, single-session duration, and weekly exercise volume did not show statistically detectable associations (*p* > 0.05). Given the limited number of contributing studies/effect sizes and the examination of multiple moderators, these findings should be interpreted cautiously (Figure 4).

Four studies, comprising four effect sizes and 740 participants, were included in the meta-analysis of estrone (E_1_). Analysis using a DerSimonian–Laird random-effects model with Hartung–Knapp-adjusted confidence intervals indicated that E_1_ levels decreased following aerobic exercise interventions (Hedges’ g = −0.19; 95% CI: −0.33 to −0.04; 95% PI: −0.42 to 0.05; *p* < 0.05). Although fewer studies were available for E_1_ than for estradiol, the direction of effects was relatively consistent across studies. However, the prediction interval included the null value (95% PI: −0.42 to 0.05), indicating uncertainty regarding the magnitude of effects that may be observed in future studies.

#### 3.4.2. Effects of Aerobic Exercise on SHBG and Free Estradiol

Sex-hormone-binding globulin (SHBG) was analyzed as a key marker of estrogen-binding status. A total of 9 studies, comprising 10 effect sizes and 1024 participants, were included in the SHBG analysis. The pooled estimate favored higher SHBG levels following aerobic exercise interventions (Hedges’ g = 0.49; 95% CI: 0.06 to 0.92; 95% PI: −1.01 to 1.99; *p* < 0.05). However, the 95% CI was wide, encompassing effects ranging from close to the null to a large increase, and between-study heterogeneity was considerable (I^2^ = 83.7%). In addition, the prediction interval crossed the null value, indicating that both the magnitude and direction of SHBG responses may vary substantially across future comparable populations and settings. Accordingly, the pooled estimate of g = 0.49 should be interpreted as an average across highly heterogeneous studies rather than as evidence of a stable or broadly generalizable increase in SHBG.

Subgroup analyses suggested that assay platform and exercise type may be associated with differences in SHBG effects (*p* < 0.05). In the health-status subgroup analysis, otherwise healthy women with overweight or obesity were analyzed separately from women with specific clinical conditions, including PCOS and breast cancer. No statistically significant difference was observed among the health-status subgroups (*p* for subgroup difference = 0.2803; Supplementary Material SE). Fasting status and exercise intensity also did not show statistically significant subgroup differences (*p* > 0.05). Menopausal status also did not show a statistically significant subgroup difference (*p* > 0.05). Because several subgroup comparisons were based on a limited number of studies, these findings should be regarded as exploratory and should not be interpreted as evidence of clinically equivalent responses across subgroups.

For free estradiol (Free E_2_), meta-analysis of three effect sizes involving 695 participants yielded a pooled estimate favoring lower Free E_2_ levels following aerobic exercise interventions (Hedges’ g = −0.18; 95% CI: −0.33 to −0.04; 95% PI: −0.51 to 0.14; *p* < 0.05). However, the prediction interval included the null value, indicating uncertainty regarding the magnitude and consistency of the effect that may be observed in future studies. Moreover, only three effect sizes contributed to this analysis, limiting confidence in the precision and generalizability of the pooled estimate. Therefore, the observed reduction in Free E_2_ should be considered preliminary and interpreted cautiously pending confirmation in additional studies.

#### 3.4.3. Effects of Aerobic Exercise on Body Composition-Related Outcomes

This study further examined the effects of aerobic exercise on body composition-related outcomes. Nine studies, comprising 10 effect sizes and 784 participants, were included in the analysis of body weight. The pooled estimate favored greater reductions in body weight following aerobic exercise interventions than in control conditions (MD = −3.06; 95% CI: −4.60 to −1.51; 95% PI: −6.95 to 0.83; *p* < 0.05). Moderate between-study heterogeneity was observed (I^2^ = 44.2%), indicating variability in the magnitude of weight change across studies. In addition, the prediction interval included the null value, indicating uncertainty in the distribution of effects across studies despite the average pooled estimate favoring weight reduction.

Nine studies, comprising 10 effect sizes and 396 participants, were included in the analysis of body mass index (BMI). The pooled estimate favored lower BMI values following aerobic exercise interventions (MD = −1.48; 95% CI: −2.91 to −0.06; 95% PI: −6.42 to 3.45; *p* < 0.05). However, between-study heterogeneity was considerable (I^2^ = 91.5%), and the prediction interval crossed the null value and extended in both directions. Thus, although the average pooled estimate indicated a reduction in BMI, the magnitude of the effect varied substantially across the included studies, and the pooled MD of −1.48 should be interpreted cautiously.

In addition, four studies, comprising five effect sizes and 202 participants, were included in the analysis of waist circumference, and four studies, comprising five effect sizes and 283 participants, were included in the analysis of total fat mass. The pooled estimates indicated reductions in both waist circumference (MD = −2.50; 95% CI: −3.59 to −1.41; 95% PI: −4.04 to −0.96; *p* < 0.05) and total fat mass (MD = −2.30; 95% CI: −3.48 to −1.12; 95% PI: −4.48 to −0.12; *p* < 0.05).

#### 3.4.4. Sensitivity Analyses and Publication Bias

Leave-one-out sensitivity analyses showed that the direction of the overall effects for E_2_, E_1_, Free E_2_, SHBG, and body composition-related outcomes remained largely unchanged after the sequential exclusion of individual studies, indicating that no single study substantially altered the overall direction of the pooled estimates.

Additional sensitivity analyses were performed by varying the assumed pre–post correlation coefficient (r = 0.2, 0.5, and 0.8) for outcomes in which the SD of the change score required imputation. The direction of the pooled effects remained unchanged across all examined correlation assumptions. The findings for waist circumference, body weight, E_1_, E_2_, SHBG, and total fat mass remained statistically significant across the three values of r. The BMI estimate also remained directionally consistent, although statistical significance was borderline when r = 0.8 (*p* = 0.05). For Free E_2_, the pooled effect remained in the same direction across all correlation assumptions, but statistical significance varied according to the assumed correlation coefficient (*p* = 0.054 at r = 0.2, *p* = 0.015 at r = 0.5, and *p* < 0.001 at r = 0.8), indicating some sensitivity of this outcome to the covariance assumption. Detailed results are provided in Supplementary Material SH.

Regarding publication bias, Egger’s test did not indicate evidence of a small-study effect for either E_2_ (*p* = 0.75) or SHBG (*p* = 0.16). Because only a limited number of studies were available for outcomes such as E_1_, Free E_2_, waist circumference, and total fat mass, formal tests for publication bias had limited interpretability for these outcomes. These findings should therefore be interpreted cautiously in light of the small number of contributing studies and between-study heterogeneity.

Additional sensitivity analyses were conducted by excluding the four studies judged to have an overall high risk of bias. The pooled effects remained directionally consistent for the main outcomes, including E_2_, E_1_, SHBG, body weight, and total fat mass. However, the statistical significance of BMI was attenuated after exclusion of high-risk studies. Detailed results are presented in Supplementary Material SI.

#### 3.4.5. Quality of Evidence

The GRADE assessment indicated that the certainty of evidence ranged from moderate to very low across the included outcomes, with most outcomes rated as low or very low. Specifically, the certainty of evidence was very low for SHBG, low for E_2_, E_1_, Free E_2_, BMI, waist circumference, and total fat mass, and moderate for body weight. The main reasons for downgrading included substantial unexplained between-study heterogeneity for selected outcomes, risk of bias in some studies, limited sample sizes, and imprecision of the effect estimates. Overall, current evidence suggests that aerobic exercise may improve endogenous estrogen-related markers and body composition-related outcomes in women with overweight or obesity. However, given the limited certainty of the evidence, these conclusions should be interpreted cautiously as suggestive rather than definitive findings.

## 4. Discussion

To the best of our knowledge, this study is among the first systematic reviews and meta-analyses to examine the effects of aerobic exercise on circulating estrogen-related markers and body composition in women with overweight or obesity. After applying the Hartung–Knapp adjustment within a random-effects model, aerobic exercise interventions were associated with lower E_1_ and E_2_ levels, higher SHBG levels, and reductions in body composition-related measures, including body weight, BMI, waist circumference, and total fat mass. A lower Free E_2_ level was also observed; however, this finding was based on only three effect sizes, the prediction interval crossed the null value, and the GRADE certainty of evidence was low, and should therefore be considered preliminary. These findings indicate concurrent changes in circulating estrogen-related markers, SHBG, and body composition-related outcomes following aerobic exercise interventions. However, the available study-level evidence does not establish causal or mediating relationships among these outcomes, nor does it demonstrate corresponding changes in tissue estrogen exposure or estrogen signalling. Between-study heterogeneity was moderate for E_2_ and remained considerable for SHBG, and the included studies varied in population characteristics, menopausal status, underlying health conditions, exercise prescriptions, and methodological features. In addition, the GRADE certainty of evidence ranged from moderate to very low across outcomes, with most outcomes rated as low or very low. Accordingly, the hormonal findings should be interpreted as preliminary and hypothesis-generating, particularly for Free E_2_, whereas the body composition findings should be considered separately and should not be used as causal evidence for the observed changes in circulating estrogen-related markers.

### 4.1. Effects of Aerobic Exercise on the Endogenous Estrogenic Environment and Potential Regulatory Mechanisms

The meta-analysis indicated that aerobic exercise interventions were associated with reduced estradiol (E_2_) levels in women with overweight or obesity, while estrone (E_1_) levels also showed an overall downward pattern. For E_2_, between-study heterogeneity was moderate after the updated analysis. This finding is consistent with the study by Ennour-Idrissi et al. [28], which showed that physical activity interventions reduced both total and free estradiol levels in women, suggesting that exercise may reduce circulating estrogen exposure. Compared with estradiol, estrone has weaker estrogenic activity; however, as an important form of peripheral estrogen in women, it may serve as a precursor or reserve source for estradiol [65,66,67]. The concurrent decreases in E_1_ and E_2_ therefore suggest that aerobic exercise may be associated with changes across multiple circulating estrogen-related markers rather than with a single hormone in isolation. These changes may also be compatible with broader alterations in estrogen-related metabolic processes, although the underlying physiological pathways remain uncertain.

From the perspective of the obesity-related endocrine phenotype, such changes are biologically plausible. Adipose tissue is both a major target organ for estrogen action and an important site of peripheral estrogen production, particularly in postmenopausal women. In obesity, adipose tissue expansion may enhance aromatase activity and increase the peripheral conversion of androgens into estrogens. Aerobic exercise, through increased energy expenditure, fat mobilization, and improvements in body composition, may provide a physiological context in which circulating estrogen-related markers also change. In the present study, reductions in body weight, BMI, waist circumference, and total fat mass occurred alongside reductions in E_1_ and E_2_, suggesting a possible relationship between changes in body composition and circulating estrogen profiles. Exploratory meta-regression suggested nominal associations of baseline BMI, changes in BMI, and changes in body weight with E_2_ responses, whereas exercise frequency, intervention duration, single-session duration, and weekly exercise volume were not statistically associated with E_2_ changes.

Because these associations were derived from study-level data, they should be regarded as exploratory and biologically plausible rather than as evidence of a specific mediating pathway. Subgroup analyses did not show statistically significant differences in E_2_ effects according to fasting status, health status, exercise intensity, measurement method, exercise type, or menopausal status. The observed variation in circulating E_1_ and E_2_ may also partly reflect methodological differences across studies, including assay methods, blood sampling timing, fasting status, menstrual cycle control, and reporting of hormonal medication use, in addition to factors such as dietary control, changes in energy intake, and baseline obesity severity. These differences may influence measured hormone concentrations and contribute to between-study variability. Accordingly, the reductions in E_1_ and E_2_ are best interpreted as circulating hormonal changes observed alongside improvements in body composition, with the mechanisms and sources of variability underlying these responses remaining to be clarified.

### 4.2. SHBG as a Bridging Marker Linking Obesity-Related Metabolic Status and Estrogen Bioavailability

The pooled analyses suggested higher circulating sex-hormone-binding globulin (SHBG) levels following aerobic exercise interventions. A lower pooled free estradiol level was also observed; however, this finding was based on only three effect sizes, the prediction interval crossed the null value, and the GRADE certainty of evidence was low. Therefore, the finding for free estradiol should be considered preliminary. Free estradiol represents the circulating unbound fraction of estradiol and is influenced by total E_2_, SHBG, and albumin. Therefore, the concurrent changes in SHBG and free estradiol may be consistent with alterations in circulating hormone-binding status. However, these findings should not be interpreted as direct evidence of reduced tissue estrogen exposure, altered estrogen signalling, or reduced adipose-derived estrogen production. In addition, the present study-level data cannot determine whether changes in SHBG mediate changes in free estradiol or whether changes in body composition mediate the observed changes in circulating estrogen-related markers. Accordingly, SHBG and free estradiol should be interpreted as complementary circulating markers rather than as evidence of a confirmed mechanistic pathway.

These findings can nevertheless be considered within the broader metabolic and endocrine context of obesity. In women with overweight or obesity, excess adiposity is often accompanied by insulin resistance and hyperinsulinemia, and elevated insulin concentrations may suppress hepatic SHBG synthesis, thereby influencing the circulating free fraction of sex hormones [68,69,70,71]. Thus, the concurrent changes in body composition and circulating hormone-related markers may reflect parallel responses to aerobic exercise, but the available aggregate data do not establish a temporal sequence, causal relationship, or mediating pathway among changes in adiposity, SHBG, and circulating estrogen measures.

However, the findings for SHBG and free estradiol should be interpreted with caution for different reasons. The SHBG analysis showed considerable between-study heterogeneity, although Egger’s test did not indicate evidence of a small-study effect, whereas the evidence for free estradiol was based on only three effect sizes and had low certainty of evidence. In addition, sensitivity analyses showed that although the direction of the pooled effect for free estradiol remained consistent across assumed pre–post correlation coefficients (r = 0.2, 0.5, and 0.8), its statistical significance varied according to the assumed correlation, indicating some sensitivity to the covariance assumption. Together with the prediction interval crossing the null value, these findings further indicate that the observed reduction in free estradiol remains preliminary and requires confirmation in larger trials. The variability in these findings may reflect both clinical and methodological heterogeneity across studies. In the SHBG analysis, subgroup differences were observed according to assay platform and exercise type, suggesting that measurement methods and exercise modality may contribute to variation in the estimated effects. However, these subgroup findings were based on a limited number of studies and should therefore be regarded as exploratory. Differences in blood sampling timing, menstrual cycle control, and reporting of hormonal medication use may have introduced additional variability in measured SHBG and free estradiol concentrations. In parallel, although all participants had overweight or obesity, the included studies differed in obesity severity, menopausal status, and baseline health conditions, and some involved specific clinical populations such as women with polycystic ovary syndrome, breast cancer, or infertility. These methodological and clinical differences may influence baseline SHBG and free estradiol levels as well as their observed responses to aerobic exercise. Therefore, while the concurrent higher SHBG and lower free estradiol estimates are consistent with a potential role of hormone-binding status in exercise-related changes in estrogen bioavailability, the current study does not establish that increased SHBG mediates the relationship between improvements in body composition and reductions in free estradiol [72,73,74,75,76,77].

### 4.3. Menopausal Status and Clinical Phenotypes May Influence the Interpretation of Results

Menopausal status may be an important factor influencing the relationship between aerobic exercise and changes in endogenous estrogens. Subgroup analysis showed that E_2_ levels tended to decrease in both premenopausal and postmenopausal women following aerobic exercise interventions. However, the difference in effects between these groups was not statistically significant, suggesting that current evidence is insufficient to confirm that menopausal status modifies the effect of aerobic exercise on E_2_ levels. Nevertheless, given the limited number of included studies, unequal subgroup sample sizes, and inadequate control for menopausal status, menstrual cycle phase, and blood sampling timing in some studies, the potential influence of menopausal status on intervention effects cannot be excluded.

From a physiological perspective, changes in E_2_ levels among premenopausal, perimenopausal, and postmenopausal women should not be interpreted through simple pooled comparisons. In postmenopausal women, ovarian estrogen secretion declines markedly, making adipose tissue-derived peripheral estrogen production relatively more important. Accordingly, changes in E_1_, E_2_, and SHBG in postmenopausal women with overweight or obesity should be interpreted in the context of the greater relative contribution of adipose tissue-derived peripheral estrogen production and changes in estrogen bioavailability. In contrast, E_2_ levels in premenopausal women are strongly influenced by menstrual cycle phase, ovulatory status, energy availability, and blood sampling timing. Thus, a post-exercise decrease in E_2_ does not necessarily indicate a beneficial endocrine change and may instead reflect short-term energy deficiency or menstrual cycle-related hormonal fluctuations. Peri-menopausal women are characterized by declining ovarian function and marked hormonal variability, making their E_2_ levels particularly sensitive to menstrual irregularity, ovulatory status, and sampling timing [78,79]. Future studies should therefore conduct more detailed stratified analyses by menopausal status and use standardized or highly comparable sampling and assay protocols.

Differences in clinical phenotypes also warrant attention. Although the study population consisted entirely of women with overweight or obesity, it included otherwise healthy women as well as women with polycystic ovary syndrome (PCOS), breast cancer, infertility, and other specific clinical conditions. Subgroup analysis did not show a statistically significant difference in E_2_ effects among health-status subgroups. PCOS is characterized by insulin resistance, hyperandrogenism, and ovulatory dysfunction; therefore, although increased SHBG and reduced free sex hormone levels may have important metabolic implications in this population, PCOS should not be considered equivalent to general obesity. Similarly, although estrogen exposure is associated with disease risk and prognosis in patients with breast cancer, the findings of this study should not be interpreted as evidence that aerobic exercise has a definitive anti-tumor effect. In infertile women, reproductive endocrine profiles are more complex, and changes in E_2_ or free estradiol levels cannot be simply classified as beneficial or harmful. Therefore, the current findings are best interpreted as reflecting general trends among women with overweight or obesity and should not be directly extrapolated to all clinical subgroups.

### 4.4. Exercise Prescription and Dose–Response: Evidence Remains Hypothesis-Generating

Exploratory meta-regression suggested nominal study-level associations of baseline BMI and changes in BMI and body weight with changes in E_2_ levels, whereas exercise frequency, intervention duration, single-session duration, and weekly exercise volume were not statistically associated with E_2_ changes. The ACSM exercise prescription guidelines indicate that exercise-induced adaptations in body composition and endocrine function may depend on an adequate cumulative exercise stimulus [80]. Similarly, previous evidence has suggested that exercise frequency and weekly exercise volume may influence energy expenditure, body weight, fat mass, adipose tissue function, and endocrine responses [81]. However, these considerations provide a theoretical context rather than direct support for a dose–response relationship in the present analysis.

Accordingly, the current meta-regression findings should not be interpreted as evidence of a definitive dose–response relationship, nor can they be used to determine the optimal aerobic exercise frequency, intensity, duration, or weekly volume for modifying circulating estrogen-related markers. Substantial collinearity may exist among exercise frequency, session duration, intensity, intervention duration, and weekly exercise volume. In addition, the limited number of contributing studies/effect sizes and the use of aggregate study-level data prevented adequate adjustment for individual-level factors such as energy intake, exercise adherence, changes in body fat, insulin resistance, and menopausal status. Therefore, the observed study-level associations of baseline BMI and changes in BMI and body weight with E_2_ effect estimates should be regarded as exploratory and hypothesis-generating rather than as evidence of mediation or as a direct basis for clinical exercise prescription. Future adequately powered randomized controlled trials and individual participant data meta-analyses are needed to clarify whether exercise dose and changes in body composition are independently associated with changes in circulating E_2_.

### 4.5. Clinical Implications and Future Research Directions

This study suggests that aerobic exercise may represent a useful non-pharmacological strategy for improving body composition in women with overweight or obesity and may also be associated with changes in circulating estrogen-related markers. In the present review, changes in body composition and SHBG were observed concurrently, alongside a reduction in free estradiol; however, the latter finding should be interpreted cautiously given the limited number of contributing effect sizes and the prediction interval crossing the null value. These findings may reflect related metabolic and endocrine responses to aerobic exercise. However, the available evidence does not allow the direction or causal relationships among these changes to be determined. From a clinical perspective, current evidence supports regular aerobic exercise as an important component of weight management, fat mass reduction, and metabolic health improvement in women with overweight or obesity. Importantly, the evidence for body composition outcomes should be distinguished from the evidence for circulating-estrogen-related outcomes. Although aerobic exercise has an established role in weight management and body composition improvement, the evidence regarding changes in E_1_, E_2_, Free E_2_, and SHBG in the present review is more uncertain because of between-study heterogeneity, methodological variability, and generally low or very low certainty of evidence. This uncertainty is particularly relevant to Free E_2_, for which the available evidence remains preliminary. Accordingly, improvements in body composition should not be considered evidence of a causal explanation for the observed hormonal changes. However, the evidence remains insufficient to recommend aerobic exercise as a specific intervention for treating estrogen-related diseases or for precisely modifying reproductive endocrine function. Current evidence is also insufficient to determine the optimal exercise prescription or to identify specific subgroups that may show greater endocrine responses to aerobic exercise.

This study also suggests that future evaluations of aerobic exercise interventions should extend beyond body weight and BMI. Researchers should report waist circumference, fat mass, SHBG, Free E_2_, estrone (E_1_), estradiol (E_2_), and markers of insulin resistance or inflammation to provide a more comprehensive characterization of obesity-related endocrine risk profiles. Future randomized controlled trials should also: (1) stratify participants by premenopausal, peri-menopausal, and postmenopausal status; (2) clearly document menstrual cycle phase, blood sampling time, fasting status, and the use of hormone replacement therapy or oral contraceptives; (3) use standardized or high-sensitivity hormone assay methods; (4) fully report exercise frequency, intensity, duration, type, intervention period, weekly exercise volume, and adherence; (5) distinguish the independent effects of aerobic exercise from those of co-interventions, such as dietary control, energy restriction, or other lifestyle changes; and (6) simultaneously assess body composition, insulin sensitivity, SHBG, and free estrogen to determine whether improvements in body composition and changes in SHBG mediate the effects of aerobic exercise on estrogen bioavailability. For evidence synthesis, future studies could use individual participant data meta-analyses to further clarify differential responses according to menopausal status, obesity severity, metabolic phenotype, and exercise prescription.

### 4.6. Strengths and Limitations

This study has several strengths. First, it focused specifically on women with overweight or obesity, a population characterized by distinct adipose tissue and endocrine–metabolic profiles, thereby distinguishing it from broader exercise intervention studies involving women. Second, this study considered not only total estrogen markers, such as E_1_ and E_2_, but also sex-hormone-binding globulin (SHBG), Free E_2_, and body composition outcomes, providing a more integrated assessment of the potential effects of aerobic exercise on both estrogen exposure and bioavailability. Third, by including randomized controlled trials and performing multiple sensitivity analyses, including leave-one-out analyses, alternative pre–post correlation assumptions, and exclusion of studies with high risk of bias, together with subgroup analyses, exploratory meta-regression, publication bias assessments, and GRADE evidence certainty evaluations, this study enhanced the transparency and robustness of result interpretation.

Several limitations should also be acknowledged. First, some included studies did not directly report the standard deviation (SD) of change scores from baseline to post-intervention. For these studies, the SD of change was estimated using statistical formulas and an assumed pre–post correlation coefficient. Although r = 0.5 was used in the primary analysis, sensitivity analyses using r = 0.2 and r = 0.8 showed that the direction of the pooled effects remained consistent across the examined outcomes. Nevertheless, the magnitude and precision of some estimates varied according to the assumed correlation, and the statistical significance of Free E_2_ was sensitive to this assumption. In addition, the Free E_2_ analysis was based on only three effect sizes, and its prediction interval crossed the null value; therefore, this finding should be considered preliminary. Therefore, uncertainty associated with imputation of change-score variances cannot be completely excluded. Second, substantial heterogeneity existed in participant characteristics across the included studies, including differences in age, BMI, menopausal status, baseline health status, obesity severity, and underlying clinical conditions. These factors may influence endogenous estrogen levels, SHBG, Free E_2_, and physiological responses to aerobic exercise. Third, exercise prescriptions varied considerably across studies. Differences in exercise type, intensity, frequency, session duration, intervention duration, and weekly exercise volume limited the ability to determine an optimal aerobic exercise prescription based on the current evidence. Fourth, the included studies differed in hormone assay methods, blood sampling timing, fasting status, menstrual cycle control, and reporting of hormonal medication use. These methodological inconsistencies may have increased measurement error in hormonal outcomes and contributed to between-study heterogeneity. Fifth, the certainty of evidence ranged from moderate to very low across outcomes. Risk of bias contributed to the downgrading of evidence certainty for several outcomes, as four of the 15 included trials (26.7%) were judged to have an overall high risk of bias. However, sensitivity analyses excluding these studies showed that the direction of effects remained consistent for most outcomes, although the statistical significance of BMI was attenuated. The certainty was very low for SHBG, low for E_2_, E_1_, Free E_2_, BMI, waist circumference, and total fat mass, and moderate for body weight. Finally, because this study primarily synthesized study-level data, it could not adequately account for individual-level variation in body composition changes, insulin resistance, energy intake, exercise adherence, menopausal status, or clinical phenotypes, nor could it directly examine causal or mediating relationships among improvements in body composition, increases in SHBG, and reductions in Free E_2_.

In summary, the evidence for body composition outcomes and circulating estrogen-related outcomes should be interpreted separately. Aerobic exercise was associated with improvements in body composition, whereas changes in E_1_, E_2_, and SHBG were also observed but were supported by a more uncertain and heterogeneous evidence base. The observed reduction in Free E_2_ should be regarded as preliminary given the limited number of effect sizes and low certainty of evidence. Although these outcomes may be biologically related, the present study-level evidence does not establish that improvements in body composition cause or mediate the observed hormonal changes. The relationships among adiposity, SHBG, and circulating estrogen-related markers should therefore be regarded as hypotheses for future mechanistic research rather than as established pathways.

## 5. Conclusions

The results of this study suggest that aerobic exercise interventions are associated with reductions in body composition-related measures and with changes in circulating estrogen-related markers in women with overweight or obesity. These two sets of findings should, however, be interpreted separately. Aerobic exercise has an established role in weight management and body composition improvement, whereas the evidence regarding changes in E_1_, E_2_, Free E_2_, and SHBG remains more uncertain and heterogeneous. The present study-level evidence does not establish that changes in body composition mediate or causally explain the observed hormonal changes. Accordingly, the estrogen-related findings should be regarded as preliminary and interpreted cautiously, particularly for outcomes supported by low or very low certainty evidence. Further high-quality randomized controlled trials with standardized interventions, harmonized hormone assessment procedures, and individual-level data are needed to clarify these relationships.

## Figures and Tables

**Table 1:** Inclusion and exclusion criteria.

	Inclusion Criteria	Exclusion Criteria
Populations (P)	Adult women aged ≥ 18 years who meet the World Health Organization criteria for overweight [25.0–29.9 kg/m^2^] or obesity [≥30.0 kg/m^2^]	Non-human studies, including animal or cell-based experiments Female minors under the age of 18
Interventions (I)	Structured, repetitive, and sustained aerobic exercise involving large muscle groups (e.g., walking, jogging, or cycling), with aerobic exercise as the primary intervention component and its independent effect distinguishable from any co-interventions.	Single-bout or acute exercise; unstructured physical activity; or mixed interventions in which aerobic exercise was not the primary intervention component or its independent effect could not be isolated.
Control (C)	No-exercise control, usual daily activities, or standard care; or a control group receiving the same co-interventions as the experimental group but without the aerobic exercise component.	Studies were excluded if aerobic exercise was combined with other structured exercise, pharmacological treatment, or therapeutic interventions, and the control group did not receive the same co-interventions, making it impossible to isolate the effects of aerobic exercise.
Outcomes (O)	Report at least one endogenous estrogen marker (e.g., E_2_, E_1_, E_3_)	No studies related to endogenous estrogens were reported.
Study (S)	Only randomized controlled trials (RCTs) were included.	1. Non-randomized controlled studies or single-group pre–post studies 2. Systematic reviews, meta-analyses, book chapters, or conference abstracts

**Table 2:** Baseline Characteristics of the Study Participants.

Author	Country	N	Age (Mean ± SD)	BMI (Mean ± SD)	Menopausal Status	Health Status	Outcomes
Tartibian et al., 2015 [50]	Iran	EXP (14)	57.1 ± 7.5	25.5 ± 3.9	Post-menopausal	Overweight	①
		CON (14)	57.2 ± 2.2	25.0 ± 3.1	Post-menopausal	Overweight	
Elsayed et al., 2022a [51]	Egypt	EXP (30)	58.99 ± 3.22	37.88 ± 1.93	Post-menopausal	Obese	①③⑤⑥
		CON (30)	58.58 ± 2.37	37.47 ± 1.39	Post-menopausal	Obese	
Friedenreich et al., 2010 [52]	Canada	EXP (160)	61.2 ± 5.4	29.1 ± 4.5	Post-menopausal	Overweight	①②③④⑤
		CON (159)	60.6 ± 5.7	29.2 ± 4.2	Post-menopausal	Overweight	
Krishnan et al., 2014 [53]	USA	EXP (18)	42–52	25.79 ± 0.92	Pre-menopausal	Overweight	①⑤⑥⑦
		CON (10)	42–52	28.01 ± 1.07	Pre-menopausal	Overweight	
Vigorito et al., 2007 [54]	Italy	EXP (45)	21.7 ± 2.3	29.3 ± 2.9	Pre-menopausal	PCOS	①③⑥⑧
		CON (45)	21.9 ± 1.9	29.4 ± 3.2	Pre-menopausal	PCOS	
Isanejad et al., 2023 [55]	Iran	EXP 1(10)	44.00 ± 9.1	26.50 ± 3.48	Mixed menopausal status	Breast cancer	①③⑤⑥⑦⑧
		EXP 2(10)	46.29 ± 6.29	28.10 ± 5.14	Mixed menopausal status	Breast cancer	①③⑤⑥⑦⑧
		CON (10)	44.9 ± 5.02	27.37 ± 4.88	Mixed menopausal status	Breast cancer	
Elsayed et al., 2022b [56]	Egypt	EXP (20)	66.03 ± 2.87	37.51 ± 1.39	Post-menopausal	Obese	①③⑤⑥
		CON (20)	65.64 ± 3.03	37.53 ± 1.16	Post-menopausal	Obese	
Ismail et al., 2022 [57]	Egypt	EXP (30)	44.26 ± 2.62	27.65 ± 1.49	Peri-menopausal	FD	①
		CON (30)	46.06 ± 2.97	27.61 ± 1.37	Peri-menopausal	FD	
McTiernan et al., 2004 [60]	USA	EXP (87)	60.7 ± 6.7	30.4 ± 4.1	Post-menopausal	Obese	①②③④
		CON (85)	60.6 ± 6.8	30.5 ± 3.7	Post-menopausal	Obese	
Jedel et al., 2011 [58]	Sweden	EXP (30)	30.2 ± 4.7	27.7 ± 6.44	Pre-menopausal	PCOS	①②③⑥
		CON (15)	30.1 ± 4.2	26.8 ± 5.56	Pre-menopausal	PCOS	
Tartibian et al., 2011 [59]	Iran	EXP (20)	61.4 ± 6.9	25.1 ± 7.1	Post-menopausal	Overweight	①
		CON (18)	58.9 ± 8.1	28.5 ± 3.7	Post-menopausal	Overweight	
Campbell et al., 2012 [61]	Canada	EXP (117)	58.1 ± 5.0	30.7 ± 3.7	Post-menopausal	Obese	①②③④⑤⑦
		CON (87)	57.4 ± 4.4	30.7 ± 3.9	Post-menopausal	Obese	
Palomba et al., 2010 [62]	Italy	EXP (32)	27.50 ± 4.95	31.26 ± 2.66	Pre-menopausal	PCOS	①③⑤⑥⑧
		CON (32)	26.50 ± 4.26	32.32 ± 3.73	Pre-menopausal	PCOS	
Yoo et al., 2010 [63]	Korea	EXP (11)	70 ± 2.7	26.6 ± 2.9	Post-menopausal	Overweight	①⑤⑥⑦
		CON (10)	71.1 ± 2.7	25.4 ± 3.0	Post-menopausal	Overweight	
Kiel et al., 2018 [64]	Norway	EXP (8)	33.1 ± 5.9	31.7 ± 4.3	Pre-menopausal	Infertile women	①⑤⑥⑧
		CON (10)	28.9 ± 2.4	31.2 ± 1.3	Pre-menopausal	Infertile women	

Table notes: EXP: Experimental group; CON: Control group; BMI: Body Mass Index; PCOS = polycystic ovary syndrome; FD: Women with functional dyspepsia; ① Estradiol; ② Estrone; ③ Sex-hormone-binding globulin; ④ Free estradiol; ⑤ Body weight; ⑥ BMI; ⑦ Total fat mass; ⑧ Waist circumference.

**Table 3:** Intervention Characteristics of the Exercise Programs in the Included Studies.

Author	Intervention	Testing Platform	Fasting Status	Intensity	Duration	Frequency (Times/wk)	Duration-Frequency (min/Week)	Session Time (min)
Tartibian et al., 2015 [50]	Jogging	IEMA	Yes	Low	16	3–6	75–270	25–45
	Usual daily activities							
Elsayed et al., 2022a [51]	Jogging	CLIA	Yes	Moderate	12	3	150	50
	Usual daily activities							
Friedenreich et al., 2010 [52]	Combined aerobic exercise	RIA	Yes	High	51	5	225	45
	Usual daily activities							
Krishnan et al., 2014 [53]	Combined aerobic exercise	UHPLC–MS/MS	Yes	High	24	6	360	60
	Usual daily activities							
Vigorito et al., 2007 [54]	Cycling	RIA	Yes	Moderate	12	3	120	40
	Usual daily activities							
Isanejad et al., 2023 [55]	Jogging	RIA	Yes	High	12	3	99	33
	Jogging	RIA	Yes	Moderate	12	3	123	41
	Usual daily activities							
Elsayed et al., 2022b [56]	Walking	CLIA	Yes	Moderate	12	3	150	50
	Usual daily activities							
Ismail et al., 2022 [57]	Walking	ELISA	NR	High	8	5	150–200	30
	Usual daily activities							
Mctiernan et al., 2004 [60]	Combined aerobic exercise	RIA	Yes	Low	51	5	225	45
	Usual daily activities							
Jedel et al., 2011 [58]	Combined aerobic exercise	GC–MS	Yes	Moderate	16	3	90	30
	Usual daily activities							
Tartibian et al., 2011 [59]	Jogging	CLIA	Yes	Low	24	3–6	75–270	25–45
	Usual daily activities							
Campbell et al., 2012 [61]	Combined aerobic exercise	RIA	Yes	High	52	5	225	45
	Usual daily activities							
Palomba et al., 2010 [62]	Cycling	NR	Yes	High	2	3	90	30
	Usual daily activities							
Yoo et al., 2010 [63]	Walking	ELISA	NR	High	12	3	180	60
	Usual daily activities							
Kiel et al., 2018 [64]	Jogging	NR	Yes	High	10	3	30–48	10–16
	Usual daily activities							

Table notes: immunoenzymometric assay (IEMA), chemiluminescent immunoassay (CLIA), radioimmunoassay (RIA), ultra-performance liquid chromatography–tandem mass spectrometry (UPLC–MS/MS), enzyme-linked immunosorbent assay (ELISA), gas chromatography–mass spectrometry (GC–MS), and not reported (NR).

## Data Availability

The original contributions presented in this study are included in the article and Supplementary Materials. Further inquiries can be directed to the corresponding author.

## Supplementary Materials

The following supporting information can be downloaded at https://www.mdpi.com/article/10.3390/metabo16100725/s1. Supplementary Material SA, PRISMA 2020 checklist; Supplementary Material SB, database search strategies; Supplementary Material SC, multilevel model validation; Supplementary Material SD, GRADE assessment of evidence regarding the effect of aerobic exercise on endogenous estrogens in overweight and obese women; Supplementary Material SE, summary of subgroup analyses; Supplementary Material SF, leave-one-out sensitivity analyses; Supplementary Material SG, influence diagnostics using Cook’s distance; Supplementary Material SH, sensitivity analyses using alternative pre–post correlation coefficients; and Supplementary Material SI, sensitivity analyses excluding studies at high risk of bias.

## Abbreviations

The following abbreviations are used in this manuscript:

ACSM	American College of Sports Medicine
AIC	Akaike information criterion
BIC	Bayesian information criterion
BMI	Body mass index
CI	Confidence interval
CLIA	Chemiluminescent immunoassay
CON	Control group
E_1_	Estrone
E_2_	Estradiol
ELISA	Enzyme-linked immunosorbent assay
EXP	Experimental group
Free E_2_	Free estradiol
GC–MS	Gas chromatography–mass spectrometry
GRADE	Grading of Recommendations Assessment, Development, and Evaluation
HRmax	Maximum heart rate
HRR	Heart rate reserve
IEMA	Immunoenzymometric assay
I^2^	I-squared statistic
MD	Mean difference
NR	Not reported
PICO	Population, Intervention, Comparator, and Outcome
PICOS	Population, Intervention, Comparator, Outcome, and Study Design
PI	Prediction interval
PCOS	Polycystic ovary syndrome
PRISMA	Preferred Reporting Items for Systematic Reviews and Meta-Analyses
PROSPERO	International Prospective Register of Systematic Reviews
RCT	Randomized controlled trial
REML	Restricted maximum likelihood
RIA	Radioimmunoassay
RoB 2	Risk of Bias 2
SD	Standard deviation
SE	Standard error
SHBG	Sex-hormone-binding globulin
SMD	Standardized mean difference
TFM	Total fat mass
UHPLC–MS/MS	Ultra-high-performance liquid chromatography–tandem mass spectrometry
UPLC–MS/MS	Ultra-performance liquid chromatography–tandem mass spectrometry
VO_2_max	Maximal oxygen uptake
WC	Waist circumference

## References

[1] Boutari C., Mantzoros C.S. (2022). A 2022 Update on the Epidemiology of Obesity and a Call to Action: As Its Twin COVID-19 Pandemic Appears to Be Receding, the Obesity and Dysmetabolism Pandemic Continues to Rage On. Metabolism.

[2] Blüher M. (2025). An Overview of Obesity-related Complications: The Epidemiological Evidence Linking Body Weight and Other Markers of Obesity to Adverse Health Outcomes. Diabetes Obes. Metab..

[3] (2017). The GBD 2015 Obesity Collaborators. Health Effects of Overweight and Obesity in 195 Countries over 25 Years. N. Engl. J. Med..

[4] Kyrgiou M., Kalliala I., Markozannes G., Gunter M.J., Paraskevaidis E., Gabra H., Martin-Hirsch P., Tsilidis K.K. (2017). Adiposity and Cancer at Major Anatomical Sites: Umbrella Review of the Literature. BMJ.

[5] Vieira-Potter V.J., Mishra G., Townsend K.L. (2026). Health of Adipose Tissue: Oestrogen Matters. Nat. Rev. Endocrinol..

[6] Stanczyk F.Z. (2024). Metabolism of Endogenous and Exogenous Estrogens in Women. J. Steroid Biochem. Mol. Biol..

[7] Thomas M.P., Potter B.V.L. (2013). The Structural Biology of Oestrogen Metabolism. J. Steroid Biochem. Mol. Biol..

[8] Hall J.M., Couse J.F., Korach K.S. (2001). The Multifaceted Mechanisms of Estradiol and Estrogen Receptor Signaling. J. Biol. Chem..

[9] Orisaka M., Miyazaki Y., Shirafuji A., Tamamura C., Tsuyoshi H., Tsang B.K., Yoshida Y. (2021). The Role of Pituitary Gonadotropins and Intraovarian Regulators in Follicle Development: A Mini-review. Reprod. Med. Biol..

[10] Mauvais-Jarvis F., Clegg D.J., Hevener A.L. (2013). The Role of Estrogens in Control of Energy Balance and Glucose Homeostasis. Endocr. Rev..

[11] Kuryłowicz A. (2023). Estrogens in Adipose Tissue Physiology and Obesity-Related Dysfunction. Biomedicines.

[12] Winters S.J., Scoggins C.R., Appiah D., Ghooray D.T. (2020). The Hepatic Lipidome and HNF4α and SHBG Expression in Human Liver. Endocr. Connect..

[13] Verhoog N.J.D., Africander D., Storbeck K.-H. (2026). Sex Steroids, SHBG and Type 2 Diabetes in Women: What Do We Really Know?. Diabetologia.

[14] Zahid H., Simpson E.R., Brown K.A. (2016). Inflammation, Dysregulated Metabolism and Aromatase in Obesity and Breast Cancer. Curr. Opin. Pharmacol..

[15] Brown K.A. (2021). Metabolic Pathways in Obesity-Related Breast Cancer. Nat. Rev. Endocrinol..

[16] Shufelt C.L., Brown V., Carpenter J.S., Chism L.A., Faubion S.S., Joffe H., Kling J.M., Soares C.N., Thurston R.C. (2023). The 2023 Nonhormone Therapy Position Statement of The North American Menopause Society. Menopause.

[17] Lumsden M.A., Dekkers O.M., Faubion S.S., Lindén Hirschberg A., Jayasena C.N., Lambrinoudaki I., Louwers Y., Pinkerton J.V., Sojat A.S., Van Hulsteijn L. (2025). European Society of Endocrinology Clinical Practice Guideline for Evaluation and Management of Menopause and the Perimenopause. Eur. J. Endocrinol..

[18] Faubion S.S., Crandall C.J., Davis L., El Khoudary S.R., Hodis H.N., Lobo R.A., Maki P.M., Manson J.E., Pinkerton J.V., Santoro N.F. (2022). The 2022 Hormone Therapy Position Statement of The North American Menopause Society. Menopause.

[19] Grossman D.C., Curry S.J., Owens D.K., Barry M.J., Davidson K.W., Doubeni C.A., Epling J.W., Kemper A.R., Krist A.H., US Preventive Services Task Force (2017). Hormone Therapy for the Primary Prevention of Chronic Conditions in Postmenopausal Women: US Preventive Services Task Force Recommendation Statement. JAMA.

[20] Main C., Knight B., Moxham T., Gabriel Sanchez R., Sanchez Gomez L.M., Roqué I., Figuls M., Bonfill Cosp X., The Cochrane Collaboration (2013). Hormone Therapy for Preventing Cardiovascular Disease in Post-Menopausal Women. Cochrane Database of Systematic Reviews.

[21] Duggan C., Tapsoba J.D.D., Stanczyk F., Wang C.Y., Schubert K.F., McTiernan A. (2019). Long-Term Weight Loss Maintenance, Sex Steroid Hormones, and Sex Hormone-Binding Globulin. Menopause.

[22] Wiggs A.G., Chandler J.K., Aktas A., Sumner S.J., Stewart D.A. (2021). The Effects of Diet and Exercise on Endogenous Estrogens and Subsequent Breast Cancer Risk in Postmenopausal Women. Front. Endocrinol..

[23] Jaguri A., Al Thani A.A., Elrayess M.A. (2023). Exercise Metabolome: Insights for Health and Performance. Metabolites.

[24] Monsalve F.A., Delgado-López F., Fernández-Tapia B., González D.R. (2023). Adipose Tissue, Non-Communicable Diseases, and Physical Exercise: An Imperfect Triangle. Int. J. Mol. Sci..

[25] Liedtke S., Schmidt M.E., Vrieling A., Lukanova A., Becker S., Kaaks R., Zaineddin A.K., Buck K., Benner A., Chang-Claude J. (2012). Postmenopausal Sex Hormones in Relation to Body Fat Distribution. Obesity.

[26] Jayedi A., Soltani S., Emadi A., Zargar M.-S., Najafi A. (2024). Aerobic Exercise and Weight Loss in Adults: A Systematic Review and Dose-Response Meta-Analysis. JAMA Netw. Open.

[27] Oppert J., Bellicha A., Van Baak M.A., Battista F., Beaulieu K., Blundell J.E., Carraça E.V., Encantado J., Ermolao A., Pramono A. (2021). Exercise Training in the Management of Overweight and Obesity in Adults: Synthesis of the Evidence and Recommendations from the European Association for the Study of Obesity Physical Activity Working Group. Obes. Rev..

[28] Ennour-Idrissi K., Maunsell E., Diorio C. (2015). Effect of Physical Activity on Sex Hormones in Women: A Systematic Review and Meta-Analysis of Randomized Controlled Trials. Breast Cancer Res..

[29] Smith A.J., Phipps W.R., Arikawa A.Y., O’Dougherty M., Kaufman B., Thomas W., Schmitz K.H., Kurzer M.S. (2011). Effects of Aerobic Exercise on Premenopausal Sex Hormone Levels: Results of the WISER Study, a Randomized Clinical Trial in Healthy, Sedentary, Eumenorrheic Women. Cancer Epidemiol. Biomark. Prev..

[30] Wang X., Simpson E.R., Brown K.A. (2015). Aromatase Overexpression in Dysfunctional Adipose Tissue Links Obesity to Postmenopausal Breast Cancer. J. Steroid Biochem. Mol. Biol..

[31] De Roon M., May A.M., McTiernan A., Scholten R.J.P.M., Peeters P.H.M., Friedenreich C.M., Monninkhof E.M. (2018). Effect of Exercise and/or Reduced Calorie Dietary Interventions on Breast Cancer-Related Endogenous Sex Hormones in Healthy Postmenopausal Women. Breast Cancer Res..

[32] Friedenreich C.M., Neilson H.K., Woolcott C.G., Wang Q., Yasui Y., Brant R.F., Stanczyk F.Z., Campbell K.L., Courneya K.S. (2011). Mediators and Moderators of the Effects of a Year-Long Exercise Intervention on Endogenous Sex Hormones in Postmenopausal Women. Cancer Causes Control.

[33] Page M.J., McKenzie J.E., Bossuyt P.M., Boutron I., Hoffmann T.C., Mulrow C.D., Shamseer L., Tetzlaff J.M., Akl E.A., Brennan S.E. (2021). The PRISMA 2020 Statement: An Updated Guideline for Reporting Systematic Reviews. BMJ.

[34] Garber C.E., Blissmer B., Deschenes M.R., Franklin B.A., Lamonte M.J., Lee I.-M., Nieman D.C., Swain D.P. (2011). Quantity and Quality of Exercise for Developing and Maintaining Cardiorespiratory, Musculoskeletal, and Neuromotor Fitness in Apparently Healthy Adults: Guidance for Prescribing Exercise. Med. Sci. Sports Exerc..

[35] Armstrong T., Bull F. (2006). Development of the World Health Organization Global Physical Activity Questionnaire (GPAQ). J. Public Health.

[36] Cumpston M., Li T., Page M.J., Chandler J., Welch V.A., Higgins J.P., Thomas J. (2019). Updated Guidance for Trusted Systematic Reviews: A New Edition of the Cochrane Handbook for Systematic Reviews of Interventions. Cochrane Database Syst. Rev..

[37] Cochrane Collaboration (2008). Cochrane Handbook for Systematic Reviews of Interventions.

[38] Akaike H. (1978). A New Look at the Statistical Model Identification. Automatica.

[39] DerSimonian R., Laird N. (1986). Meta-Analysis in Clinical Trials. Control. Clin. Trials.

[40] Röver C., Knapp G., Friede T. (2015). Hartung-Knapp-Sidik-Jonkman Approach and Its Modification for Random-Effects Meta-Analysis with Few Studies. BMC Med. Res. Methodol..

[41] Takeshima N., Sozu T., Tajika A., Ogawa Y., Hayasaka Y., Furukawa T.A. (2014). Which Is More Generalizable, Powerful and Interpretable in Meta-Analyses, Mean Difference or Standardized Mean Difference?. BMC Med. Res. Methodol..

[42] Deeks J.J., Higgins J.P., Altman D.G., on behalf of the Cochrane Statistical Methods Group (2019). Analysing data and undertaking meta-analyses. Cochrane Handbook for Systematic Reviews of Interventions.

[43] Nagashima K., Noma H., Furukawa T.A. (2019). Prediction Intervals for Random-Effects Meta-Analysis: A Confidence Distribution Approach. Stat. Methods Med. Res..

[44] Nakagawa S., Noble D.W.A., Senior A.M., Lagisz M. (2017). Meta-Evaluation of Meta-Analysis: Ten Appraisal Questions for Biologists. BMC Biol..

[45] Ruppar T. (2020). Meta-Analysis: How to Quantify and Explain Heterogeneity?. Eur. J. Cardiovasc. Nurs..

[46] Binney Z.O., Mansournia M.A. (2024). Methods Matter: (Mostly) Avoid Categorising Continuous Data—A Practical Guide. Br. J. Sports Med..

[47] Rico-González M., Pino-Ortega J., Clemente F., Los Arcos A. (2022). Guidelines for Performing Systematic Reviews in Sports Science. Biol. Sport.

[48] Peters J.L., Sutton A.J., Jones D.R., Abrams K.R., Rushton L. (2008). Contour-Enhanced Meta-Analysis Funnel Plots Help Distinguish Publication Bias from Other Causes of Asymmetry. J. Clin. Epidemiol..

[49] Egger M., Smith G.D., Schneider M., Minder C. (1997). Bias in Meta-Analysis Detected by a Simple, Graphical Test. BMJ.

[50] Tartibian B., FitzGerald L.Z., Azadpour N., Maleki B.H. (2015). A Randomized Controlled Study Examining the Effect of Exercise on Inflammatory Cytokine Levels in Post-Menopausal Women. Post Reprod. Health.

[51] Elsayed M.M., El Refaye G.E., Rabiee A., Abouzeid S., Elsisi H.F. (2022). Aerobic Exercise with Diet Induces Hormonal, Metabolic, and Psychological Changes in Postmenopausal Obese Women. Heliyon.

[52] Friedenreich C.M., Woolcott C.G., McTiernan A., Ballard-Barbash R., Brant R.F., Stanczyk F.Z., Terry T., Boyd N.F., Yaffe M.J., Irwin M.L. (2010). Alberta Physical Activity and Breast Cancer Prevention Trial: Sex Hormone Changes in a Year-Long Exercise Intervention Among Postmenopausal Women. J. Clin. Oncol..

[53] Krishnan S., Gustafson M.B., Campbell C., Gaikwad N.W., Keim N.L. (2014). Association between Circulating Endogenous Androgens and Insulin Sensitivity Changes with Exercise Training in Midlife Women. Menopause.

[54] Vigorito C., Giallauria F., Palomba S., Cascella T., Manguso F., Lucci R., De Lorenzo A., Tafuri D., Lombardi G., Colao A. (2007). Beneficial Effects of a Three-Month Structured Exercise Training Program on Cardiopulmonary Functional Capacity in Young Women with Polycystic Ovary Syndrome. J. Clin. Endocrinol. Metab..

[55] Isanejad A., Nazari S., Gharib B., Motlagh A.G. (2023). Comparison of the Effects of High-Intensity Interval and Moderate-Intensity Continuous Training on Inflammatory Markers, Cardiorespiratory Fitness, and Quality of Life in Breast Cancer Patients. J. Sport Health Sci..

[56] Elsayed M.M., Rabiee A., Elrefaye G.E., Elsisi H.F. (2022). Effect of Aerobic Exercise with Diet on Sex Hormones and Selected Coagulation Biomarkers in Obese Postmenopausal Women: A Randomized Clinical Trial. Eur. Rev. Med. Pharmacol. Sci..

[57] Ali Ismail A.M., Saad A.E., Fouad Abd-Elrahman N.A., Abdelhalim Elfahl A.M. (2022). Effect of Benson’s Relaxation Therapy Alone or Combined with Aerobic Exercise on Cortisol, Sleeping Quality, Estrogen, and Severity of Dyspeptic Symptoms in Perimenopausal Women with Functional Dyspepsia. Eur. Rev. Med. Pharmacol. Sci..

[58] Jedel E., Labrie F., Odén A., Holm G., Nilsson L., Janson P.O., Lind A.K., Ohlsson C., Stener-Victorin E. (2011). Impact of Electro-Acupuncture and Physical Exercise on Hyperandrogenism and Oligo/Amenorrhea in Women with Polycystic Ovary Syndrome: A Randomized Controlled Trial. Am. J. Physiol.-Endocrinol. Metab..

[59] Tartibian B., Hajizadeh Maleki B., Kanaley J., Sadeghi K. (2011). Long-Term Aerobic Exercise and Omega-3 Supplementation Modulate Osteoporosis through Inflammatory Mechanisms in Post-Menopausal Women: A Randomized, Repeated Measures Study. Nutr. Metab..

[60] McTiernan A., Tworoger S.S., Ulrich C.M., Yasui Y., Irwin M.L., Rajan K.B., Sorensen B., Rudolph R.E., Bowen D., Stanczyk F.Z. (2004). Effect of Exercise on Serum Estrogens in Postmenopausal Women: A 12-Month Randomized Clinical Trial. Cancer Res..

[61] Campbell K.L., Foster-Schubert K.E., Alfano C.M., Wang C.-C., Wang C.-Y., Duggan C.R., Mason C., Imayama I., Kong A., Xiao L. (2012). Reduced-Calorie Dietary Weight Loss, Exercise, and Sex Hormones in Postmenopausal Women: Randomized Controlled Trial. J. Clin. Oncol..

[62] Palomba S., Falbo A., Giallauria F., Russo T., Rocca M., Tolino A., Zullo F., Orio F. (2010). Six Weeks of Structured Exercise Training and Hypocaloric Diet Increases the Probability of Ovulation after Clomiphene Citrate in Overweight and Obese Patients with Polycystic Ovary Syndrome: A Randomized Controlled Trial. Hum. Reprod..

[63] Yoo E.J., Jun T.W., Hawkins S.A. (2010). The Effects of a Walking Exercise Program on Fall-Related Fitness, Bone Metabolism, and Fall-Related Psychological Factors in Elderly Women. Res. Sports Med..

[64] Kiel I.A., Lundgren K.M., Mørkved S., Kjøtrød S.B., Salvesen Ø., Romundstad L.B., Moholdt T. (2018). Women Undergoing Assisted Fertilisation and High-Intensity Interval Training: A Pilot Randomised Controlled Trial. BMJ Open Sport Exerc. Med..

[65] Hetemäki N., Robciuc A., Vihma V., Haanpää M., Hämäläinen E., Tikkanen M.J., Mikkola T.S., Savolainen-Peltonen H. (2025). Adipose Tissue Sex Steroids in Postmenopausal Women with and Without Menopausal Hormone Therapy. J. Clin. Endocrinol. Metab..

[66] Qureshi R., Picon-Ruiz M., Aurrekoetxea-Rodriguez I., Nunes De Paiva V., D’Amico M., Yoon H., Radhakrishnan R., Morata-Tarifa C., Ince T., Lippman M.E. (2020). The Major Pre- and Postmenopausal Estrogens Play Opposing Roles in Obesity-Driven Mammary Inflammation and Breast Cancer Development. Cell Metab..

[67] Davis S.R., Martinez-Garcia A., Robinson P.J., Handelsman D.J., Desai R., Wolfe R., Bell R.J. (2020). ASPREE Investigator Group Estrone Is a Strong Predictor of Circulating Estradiol in Women Age 70 Years and Older. J. Clin. Endocrinol. Metab..

[68] Senn H.-J., Goldhirsch A., Gelber R.D., Osterwalder B. (2012). Adjuvant Therapy of Primary Breast Cancer.

[69] Xing C., Zhang J., Zhao H., He B. (2022). Effect of Sex Hormone-Binding Globulin on Polycystic Ovary Syndrome: Mechanisms, Manifestations, Genetics, and Treatment. Int. J. Women’s Health.

[70] Botwood N., Hamilton-Fairley D., Kiddy D., Robinson S., Franks S. (1995). Sex Hormone-Binding Globulin and Female Reproductive Function. J. Steroid Biochem. Mol. Biol..

[71] Goldsammler M., Merhi Z., Buyuk E. (2018). Role of Hormonal and Inflammatory Alterations in Obesity-Related Reproductive Dysfunction at the Level of the Hypothalamic-Pituitary-Ovarian Axis. Reprod. Biol. Endocrinol..

[72] Hawley J.A., Hargreaves M., Joyner M.J., Zierath J.R. (2014). Integrative Biology of Exercise. Cell.

[73] Pedersen B.K., Saltin B. (2015). Exercise as medicine–Evidence for prescribing exercise as therapy in 26 different chronic diseases. Scand. J. Med. Sci. Sports.

[74] Barros R.P.A., Gustafsson J.-Å. (2011). Estrogen Receptors and the Metabolic Network. Cell Metab..

[75] Sumithran P., Prendergast L.A., Delbridge E., Purcell K., Shulkes A., Kriketos A., Proietto J. (2011). Long-Term Persistence of Hormonal Adaptations to Weight Loss. N. Engl. J. Med..

[76] Febbraio M.A., Pedersen B.K. (2026). Exercise as a Therapeutic Intervention for Long-Lasting and Chronic Diseases. Cell Metab..

[77] Lee A.A., Den Hartigh L.J. (2025). Metabolic Impact of Endogenously Produced Estrogens by Adipose Tissue in Females and Males across the Lifespan. Front. Endocrinol..

[78] Oester C., Norris D., Lovell R., Pedlar C., Bruinvels G., Kalkhoven J.T. (2026). An Overarching Conceptual Framework for Menstrual Health in Sport Research: Theory, Causality and Validation. Sports Med..

[79] Harlow S.D., Gass M., Hall J.E., Lobo R., Maki P., Rebar R.W., Sherman S., Sluss P.M., De Villiers T.J., for the Straw +10 Collaborative Group (2012). Executive Summary of the Stages of Reproductive Aging Workshop +10: Addressing the Unfinished Agenda of Staging Reproductive Aging. Climacteric.

[80] Jakicic J.M., Apovian C.M., Barr-Anderson D.J., Courcoulas A.P., Donnelly J.E., Ekkekakis P., Hopkins M., Lambert E.V., Napolitano M.A., Volpe S.L. (2024). Physical Activity and Excess Body Weight and Adiposity for Adults. American College of Sports Medicine Consensus Statement. Med. Sci. Sports Exerc..

[81] Ross R., Dagnone D., Jones P.J.H., Smith H., Paddags A., Hudson R., Janssen I. (2000). Reduction in Obesity and Related Comorbid Conditions after Diet-Induced Weight Loss or Exercise-Induced Weight Loss in Men: A Randomized, Controlled Trial. Ann. Intern. Med..

