# Supplementary material for: Effects of Aerobic Exercise on Endogenous Estrogens and Body Composition in Women with Overweight or Obesity: A Systematic Review and Meta-Analysis
Source: Metabolites. 2026 Sep 28;16(10):725. doi: 10.3390/metabo16100725 (PMC13707349; doi:10.3390/metabo16100725)
Supplement: Supplementary file 1 [file metabolites-16-00725-s001.zip › metabolites-4546674-supplementary.pdf]

Effects of Aerobic Exercise on Endogenous Estrogens and Body Composition in Women with Overweight or Obesity: A Systematic Review and Meta-Analysis

Chao Xie[1†], Mingnan Zhuang[1,2†],Jiansong Huang[3]， Yiran Guo[4*]

1 School of Sports Training, Tianjin University of Sport, Tianjin , China

2 Department of Winter Sports, Hebei Sport University, Shijiazhuang , Hebei, China

3 College of Medicine, Zhejiang University, Hangzhou, China

4 Department of Winter Sports, Hebei Sport University, Shijiazhuang , Hebei, China

† Chao Xie and Mingnan Zhuang contributed equally to this work and share first authorship.

*Correspondence to: Yiran Guo E-mail: guoyiran@hepec.edu.cn，Department of Winter Sports, Hebei Sport University, Shijiazhuang 050041, Hebei, China

Supplementary Material SA. PRISMA 2020 Checklist

Section and Topic	Item #	Checklist item	Location where item is reported
**TITLE**			
Title	1	Identify the report as a systematic review.	1
**ABSTRACT**			
Abstract	2	See the PRISMA 2020 for Abstracts checklist.	2
**INTRODUCTION**			
Rationale	3	Describe the rationale for the review in the context of existing knowledge.	3-5
Objectives	4	Provide an explicit statement of the objective(s) or question(s) the review addresses.	3-5
**METHODS**			
Eligibility criteria	5	Specify the inclusion and exclusion criteria for the review and how studies were grouped for the syntheses.	5，Table 1
Information sources	6	Specify all databases, registers, websites, organisations, reference lists and other sources searched or consulted to identify studies. Specify the date when each source was last searched or consulted.	5-7
Search strategy	7	Present the full search strategies for all databases, registers and websites, including any filters and limits used.	2;Supplementary Material B
Selection process	8	Specify the methods used to decide whether a study met the inclusion criteria of the review, including how many reviewers screened each record and each report retrieved, whether they worked independently, and if applicable, details of automation tools used in the process.	5-7
Data collection process	9	Specify the methods used to collect data from reports, including how many reviewers collected data from each report, whether they worked independently, any processes for obtaining or confirming data from study investigators, and if applicable, details of automation tools used in the process.	6-8
Data items	10a	List and define all outcomes for which data were sought. Specify whether all results that were compatible with each outcome domain in each study were sought (e.g. for all measures, time points, analyses), and if not, the methods used to decide which results to collect.	6-8
	10b	List and define all other variables for which data were sought (e.g. participant and intervention characteristics, funding sources). Describe any assumptions made about any missing or unclear information.	6-9
Study risk of bias assessment	11	Specify the methods used to assess risk of bias in the included studies, including details of the tool(s) used, how many reviewers assessed each study and whether they worked independently, and if applicable, details of automation tools used in the process.	7-9
Effect measures	12	Specify for each outcome the effect measure(s) (e.g. risk ratio, mean difference) used in the synthesis or presentation of results.	7-9
Synthesis methods	13a	Describe the processes used to decide which studies were eligible for each synthesis (e.g. tabulating the study intervention characteristics and comparing against the planned groups for each synthesis (item #5)).	7-9
	13b	Describe any methods required to prepare the data for presentation or synthesis, such as handling of missing summary statistics, or data conversions.	7-9
	13c	Describe any methods used to tabulate or visually display results of individual studies and syntheses.	7-9
	13d	Describe any methods used to synthesize results and provide a rationale for the choice(s). If meta-analysis was performed, describe the model(s), method(s) to identify the presence and extent of statistical heterogeneity, and software package(s) used.	7-9

Section and Topic	Item #	Checklist item	Location where item is reported
	13e	Describe any methods used to explore possible causes of heterogeneity among study results (e.g. subgroup analysis, meta-regression).	7-9; Supplementary Material E
	13f	Describe any sensitivity analyses conducted to assess robustness of the synthesized results.	9-10
Reporting bias assessment	14	Describe any methods used to assess risk of bias due to missing results in a synthesis (arising from reporting biases).	9-10
Certainty assessment	15	Describe any methods used to assess certainty (or confidence) in the body of evidence for an outcome.	9-10
**RESULTS**			
Study selection	16a	Describe the results of the search and selection process, from the number of records identified in the search to the number of studies included in the review, ideally using a flow diagram.	9; figure1
	16b	Cite studies that might appear to meet the inclusion criteria, but which were excluded, and explain why they were excluded.	figure1
Study characteristics	17	Cite each included study and present its characteristics.	9-10
Risk of bias in studies	18	Present assessments of risk of bias for each included study.	10； figure2
Results of individual studies	19	For all outcomes, present, for each study: (a) summary statistics for each group (where appropriate) and (b) an effect estimate and its precision (e.g. confidence/credible interval), ideally using structured tables or plots.	10-11 figure3
Results of syntheses	20a	For each synthesis, briefly summarise the characteristics and risk of bias among contributing studies.	10-11
	20b	Present results of all statistical syntheses conducted. If meta-analysis was done, present for each the summary estimate and its precision (e.g. confidence/credible interval) and measures of statistical heterogeneity. If comparing groups, describe the direction of the effect.	10-11
	20c	Present results of all investigations of possible causes of heterogeneity among study results.	10-11； Supplementary Material E
	20d	Present results of all sensitivity analyses conducted to assess the robustness of the synthesized results.	11
Reporting biases	21	Present assessments of risk of bias due to missing results (arising from reporting biases) for each synthesis assessed.	11-12
Certainty of evidence	22	Present assessments of certainty (or confidence) in the body of evidence for each outcome assessed.	11-12
**DISCUSSION**			
Discussion	23a	Provide a general interpretation of the results in the context of other evidence.	13-16
	23b	Discuss any limitations of the evidence included in the review.	16-17
	23c	Discuss any limitations of the review processes used.	17-19
	23d	Discuss implications of the results for practice, policy, and future research.	19
**OTHER INFORMATION**			
Registration and protocol	24a	Provide registration information for the review, including register name and registration number, or state that the review was not registered.	2
	24b	Indicate where the review protocol can be accessed, or state that a protocol was not prepared.	
	24c	Describe and explain any amendments to information provided at registration or in the protocol.	
Support	25	Describe sources of financial or non-financial support for the review, and the role of the funders or sponsors in the review.	19

Section and Topic	Item #	Checklist item	Location where item is reported
Competing interests	26	Declare any competing interests of review authors.	19
Availability of data, code and other materials	27	Report which of the following are publicly available and where they can be found: template data collection forms; data extracted from included studies; data used for all analyses; analytic code; any other materials used in the review.	19

Supplementary Material SB. Database Search Strategies

Summary of Web of Science Search Terms

**#1**	(women OR woman OR female OR obesity OR obese OR overweight OR "weight gain" OR "body mass index" OR BMI)	409530
**#2**	("exercise" OR "physical activity" OR "motor activity" OR "exercise training" OR "training" OR "aerobic exercise" OR "aerobic training" OR "endurance training" OR "cardiorespiratory exercise" OR "moderate-intensity exercise" OR "vigorous exercise" OR "high intensity intermittent exercise" OR "high-intensity interval exercise" OR "high-intensity interval training" OR HIIT OR "interval training" OR "continuous training" OR "exercise intervention" OR "exercise program" OR "exercise programme" OR "training intervention" OR "fitness" OR "physical fitness" OR "exercise therapy" OR "lifestyle intervention" OR "lifestyle modification" OR "lifestyle change" OR "healthy lifestyle" OR "weight loss intervention" OR "behavioral intervention" OR "behavioural intervention" OR "weight management" OR "physical exertion")	237300
**#3**	(estrogen OR estrogens OR oestrogen OR oestrogens OR estradiol OR estrone OR estriol OR "endogenous estrogen*" OR "endogenous estradiol" OR "circulating estrogen*" OR "serum estrogen*" OR "plasma estrogen*" OR "free estradiol" OR "total estradiol" OR "bioavailable estradiol" OR "estrogen metabolite*" OR "exogenous estrogen*" OR "estrogen therapy" OR "estrogen replacement therapy" OR "hormone therapy" OR "hormone replacement therapy" OR HRT OR "oral contraceptive*" OR contraceptive* OR "sex hormone-binding globulin" OR SHBG OR "free estrogen index" OR "free estradiol index" OR "sex hormone" OR "sex hormones" OR "sex steroid" OR "sex steroids" OR "steroid hormone*" OR "gonadal steroid hormone*")	39480
**#4**	**#1 AND #2 AND #3**	6537

Summary of PubMed Search Queries

#1	("Women"[MeSH Terms] OR women[Title/Abstract] OR woman[Title/Abstract] OR female[Title/Abstract] OR "Obesity"[MeSH Terms] OR "Overweight"[MeSH Terms] OR obesity[Title/Abstract] OR obese[Title/Abstract] OR overweight[Title/Abstract] OR "weight gain"[Title/Abstract] OR "body mass index"[Title/Abstract] OR BMI[Title/Abstract])	315760
#2	("Exercise"[MeSH Terms] OR "Motor Activity"[MeSH Terms] OR "Physical Fitness"[MeSH Terms] OR "Exercise Therapy"[MeSH Terms] OR "Life Style"[MeSH Terms] OR exercise[Title/Abstract] OR "physical activity"[Title/Abstract] OR "motor activity"[Title/Abstract] OR "exercise training"[Title/Abstract] OR training[Title/Abstract] OR "aerobic exercise"[Title/Abstract] OR "aerobic training"[Title/Abstract] OR "endurance training"[Title/Abstract] OR "cardiorespiratory exercise"[Title/Abstract] OR "moderate-intensity exercise"[Title/Abstract] OR "vigorous exercise"[Title/Abstract] OR "high intensity intermittent exercise"[Title/Abstract] OR "high-intensity interval exercise"[Title/Abstract] OR "high-intensity interval training"[Title/Abstract] OR HIIT[Title/Abstract] OR "interval training"[Title/Abstract] OR "continuous training"[Title/Abstract] OR "exercise intervention"[Title/Abstract] OR "exercise program"[Title/Abstract] OR "exercise programme"[Title/Abstract] OR "training intervention"[Title/Abstract] OR fitness[Title/Abstract] OR "physical fitness"[Title/Abstract] OR "exercise therapy"[Title/Abstract] OR "lifestyle intervention"[Title/Abstract] OR "lifestyle modification"[Title/Abstract] OR "lifestyle change"[Title/Abstract] OR "healthy lifestyle"[Title/Abstract] OR "weight loss intervention"[Title/Abstract] OR "behavioral intervention"[Title/Abstract] OR "behavioural intervention"[Title/Abstract] OR "weight management"[Title/Abstract] OR "physical exertion"[Title/Abstract])	153766
#3	("Estrogens"[MeSH Terms] OR "Estradiol"[MeSH Terms] OR "Sex Hormone-Binding Globulin"[MeSH Terms] OR estrogen[Title/Abstract] OR estrogens[Title/Abstract] OR oestrogen[Title/Abstract] OR oestrogens[Title/Abstract] OR estradiol[Title/Abstract] OR estrone[Title/Abstract] OR	411134

	estriol[Title/Abstract] OR "endogenous estrogen*"[Title/Abstract] OR "endogenous estradiol"[Title/Abstract] OR "circulating estrogen*"[Title/Abstract] OR "serum estrogen*"[Title/Abstract] OR "plasma estrogen*"[Title/Abstract] OR "free estradiol"[Title/Abstract] OR "total estradiol"[Title/Abstract] OR "bioavailable estradiol"[Title/Abstract] OR "estrogen metabolite*"[Title/Abstract] OR "exogenous estrogen*"[Title/Abstract] OR "estrogen therapy"[Title/Abstract] OR "estrogen replacement therapy"[Title/Abstract] OR "hormone therapy"[Title/Abstract] OR "hormone replacement therapy"[Title/Abstract] OR HRT[Title/Abstract] OR "oral contraceptive*"[Title/Abstract] OR contraceptive*[Title/Abstract] OR "sex hormone-binding globulin"[Title/Abstract] OR SHBG[Title/Abstract] OR "free estrogen index"[Title/Abstract] OR "free estradiol index"[Title/Abstract] OR "sex hormone"[Title/Abstract] OR "sex hormones"[Title/Abstract] OR "sex steroid"[Title/Abstract] OR "sex steroids"[Title/Abstract] OR "steroid hormone*"[Title/Abstract] OR "gonadal steroid hormone*"[Title/Abstract])	
#4	#1 AND #2 AND #3	5783

Summary of Cochrane Library Search Strategies

#1	[mh Women] OR [mh Obesity] OR [mh Overweight] OR (women OR woman OR female OR obesity OR obese OR overweight OR "weight gain" OR "body mass index" OR BMI):ti,ab,kw	116371
#2	[mh Exercise] OR [mh "Motor Activity"] OR [mh "Physical Fitness"] OR [mh "Exercise Therapy"] OR [mh "Life Style"] OR (exercise OR "physical activity" OR "motor activity" OR "exercise training" OR training OR "aerobic exercise" OR "aerobic training" OR "endurance training" OR "cardiorespiratory exercise" OR "moderate-intensity exercise" OR "vigorous exercise" OR "high intensity intermittent exercise" OR "high-intensity interval exercise" OR "high-intensity interval training" OR HIIT OR "interval training" OR "continuous training" OR "exercise intervention" OR "exercise program" OR "exercise programme" OR "training intervention" OR fitness OR "physical fitness" OR "exercise therapy" OR "lifestyle intervention" OR "lifestyle modification" OR "lifestyle change" OR "healthy lifestyle" OR "weight loss intervention" OR "behavioral intervention" OR "behavioural intervention" OR "weight management" OR "physical exertion"):ti,ab,kw	29776
#3	[mh Estrogens] OR [mh Estradiol] OR [mh "Sex Hormone-Binding Globulin"] OR (estrogen OR estrogens OR oestrogen OR oestrogens OR estradiol OR estrone OR estriol OR "endogenous estrogen*" OR "endogenous estradiol" OR "circulating estrogen*" OR "serum estrogen*" OR "plasma estrogen*" OR "free estradiol" OR "total estradiol" OR "bioavailable estradiol" OR "estrogen metabolite*" OR "exogenous estrogen*" OR "estrogen therapy" OR "estrogen replacement therapy" OR "hormone therapy" OR "hormone replacement therapy" OR HRT OR "oral contraceptive*" OR contraceptive* OR "sex hormone-binding globulin" OR SHBG OR "free estrogen index" OR "free estradiol index" OR "sex hormone" OR "sex hormones" OR "sex steroid" OR "sex steroids" OR "steroid hormone*" OR "gonadal steroid hormone*"):ti,ab,kw	42446
#4	#1 AND #2 AND #3	2522

Summary of Scopus Search Queries

#1	TITLE-ABS-KEY(women OR woman OR female OR obesity OR obese OR overweight OR "weight gain" OR "body mass index" OR BMI)	146793
#2	TITLE-ABS-KEY(exercise OR "physical activity" OR "motor activity" OR "exercise training" OR training OR "aerobic exercise" OR "aerobic training" OR "endurance training" OR "cardiorespiratory exercise" OR "moderate-intensity exercise" OR "vigorous exercise" OR "high intensity intermittent exercise" OR "high-intensity interval exercise" OR "high-intensity interval training" OR HIIT OR "interval training" OR "continuous training" OR "exercise intervention" OR "exercise program" OR "exercise programme" OR "training intervention" OR fitness OR "physical fitness" OR "exercise therapy" OR "lifestyle intervention" OR "lifestyle modification" OR "lifestyle change" OR "healthy lifestyle" OR "weight loss intervention" OR "behavioral intervention" OR "behavioural intervention" OR "weight management" OR "physical exertion")	349021

#3	TITLE-ABS-KEY(estrogen OR estrogens OR oestrogen OR oestrogens OR estradiol OR estrone OR estriol OR "endogenous estrogen*" OR "endogenous estradiol" OR "circulating estrogen*" OR "serum estrogen*" OR "plasma estrogen*" OR "free estradiol" OR "total estradiol" OR "bioavailable estradiol" OR "estrogen metabolite*" OR "exogenous estrogen*" OR "estrogen therapy" OR "estrogen replacement therapy" OR "hormone therapy" OR "hormone replacement therapy" OR HRT OR "oral contraceptive*" OR contraceptive* OR "sex hormone-binding globulin" OR SHBG OR "free estrogen index" OR "free estradiol index" OR "sex hormone" OR "sex hormones" OR "sex steroid" OR "sex steroids" OR "steroid hormone*" OR "gonadal steroid hormone*")	68037
#4	#1 AND #2 AND #3	3627

Summary of Embase Search Strings

#1	(women OR woman OR female OR obesity OR obese OR overweight OR "weight gain" OR "body mass index" OR BMI):ti,ab,kw	470714
#2	(exercise OR "physical activity" OR "motor activity" OR "exercise training" OR training OR "aerobic exercise" OR "aerobic training" OR "endurance training" OR "cardiorespiratory exercise" OR "moderate-intensity exercise" OR "vigorous exercise" OR "high intensity intermittent exercise" OR "high-intensity interval exercise" OR "high-intensity interval training" OR HIIT OR "interval training" OR "continuous training" OR "exercise intervention" OR "exercise program" OR "exercise programme" OR "training intervention" OR fitness OR "physical fitness" OR "exercise therapy" OR "lifestyle intervention" OR "lifestyle modification" OR "lifestyle change" OR "healthy lifestyle" OR "weight loss intervention" OR "behavioral intervention" OR "behavioural intervention" OR "weight management" OR "physical exertion"):ti,ab,kw	176267
#3	(estrogen OR estrogens OR oestrogen OR oestrogens OR estradiol OR estrone OR estriol OR "endogenous estrogen*" OR "endogenous estradiol" OR "circulating estrogen*" OR "serum estrogen*" OR "plasma estrogen*" OR "free estradiol" OR "total estradiol" OR "bioavailable estradiol" OR "estrogen metabolite*" OR "exogenous estrogen*" OR "estrogen therapy" OR "estrogen replacement therapy" OR "hormone therapy" OR "hormone replacement therapy" OR HRT OR "oral contraceptive*" OR contraceptive* OR "sex hormone-binding globulin" OR SHBG OR "free estrogen index" OR "free estradiol index" OR "sex hormone" OR "sex hormones" OR "sex steroid" OR "sex steroids" OR "steroid hormone*" OR "gonadal steroid hormone*"):ti,ab,kw	49253
#4	#1 AND #2 AND #3	2683

Supplementary Material SC. Multilevel Model Validation

Model validation								
	Df	AIC	BIC	AICc	LogLik	LRT	Pval	QE
Full	3	24.2491	26.5669	26.2491	−9.1246	NA	NA	24.4011
Reduced	2	22.2587	23.8039	23.1818	−9.1294	0.0096	0.9219	24.4011

Supplementary Material SD. GRADE assessment of evidence regarding the effect of aerobic exercise on endogenous estrogens in overweight and obese women.

	Risk of bias	Inconsistency	Indirectness	Imprecision	Publication bias	Certainty assessment
**E2**	Some concerns ↓	I^2 =38.1% ↔	Consistent comparator ↔	Serious imprecision ↓	*P*=0.75 ↔	Low
**E1**	Some concerns ↓	I^2= 0% ↔	Consistent comparator ↔	Serious imprecision ↓	*P*=0.23	Low
**SHBG**	Some concerns ↓	I^2= 83.7% ↓	Consistent comparator ↔	Serious imprecision ↓	*P*=0.16 ↔	Very low
**Free Estradiol**	Some concerns ↓	I^2 = 0% ↔	Consistent comparator ↔	Serious imprecision ↓	Not assessed	Low
**BMI**	Some concerns ↓	I^2= 91.5% ↓	Consistent comparator ↔	No serious imprecision ↔	*P*=0.50 ↔	Low
**Weight**	Some concerns ↓	I^2 = 44.2% ↔	Consistent comparator ↔	No serious imprecision ↔	*P*=0.23 ↔	Moderate
**WC**	Some concerns ↓	I^2 = 0% ↔	Consistent comparator ↔	Serious imprecision ↓	Not assessed	Low

**Total Fat Mass**	Some concerns ↓	I^2 = 0% ↔	Consistent comparator ↔	Serious imprecision ↓	Not assessed	Low

Outcome	Rationale for GRADE judgments
**E2**	**Risk of bias:** downgraded one level because the contributing trials raised concerns, particularly regarding deviations from intended interventions and missing outcome data. **Inconsistency:** not downgraded because heterogeneity was substantially reduced and was moderate (I^2 = 38.1%), with no evidence of substantial unexplained inconsistency across studies. **Imprecision:** downgraded one level because the 95% CI (Hedges' g = −0.38 to −0.06) still encompassed effects ranging from a very small to a more clearly meaningful reduction, leaving uncertainty regarding the magnitude of the effect. Statistical significance was not used as the basis for this judgment.
**E1**	**Risk of bias:** downgraded one level because of concerns regarding risk of bias in the contributing trials. **Inconsistency:** no downgrade was applied because heterogeneity was negligible (I^2 = 0%) and the direction of study-specific effects was generally consistent. **Imprecision:** downgraded one level because the 95% CI (g = −0.33 to −0.04) encompassed effects ranging from a minimal/near-null reduction to a small reduction, leaving uncertainty regarding the magnitude and potential relevance of the effect. In addition, the estimate was informed by only four study-level effect estimates, limiting the overall information available for judging precision.
**SHBG**	**Risk of bias:** downgraded one level because of concerns regarding risk of bias in the contributing trials. **Inconsistency:** downgraded one level because heterogeneity was considerable (I^2 = 83.7%), indicating substantial between-study variation in the magnitude of the effect. **Imprecision:** downgraded one level because the 95% CI (g = 0.06 to 0.92) remained relatively wide and encompassed effects ranging from close to the null to a large increase in SHBG. **Publication bias:** Egger's test did not indicate statistically significant evidence of a small-study effect (P = 0.16); however, given the limited number of independent studies, this finding should be interpreted cautiously.
**Free Estradiol**	**Risk of bias:** downgraded one level because of concerns regarding risk of bias in the contributing evidence. **Inconsistency:** no downgrade was applied because statistical heterogeneity was negligible (I^2 = 0%). **Imprecision:** downgraded one level because the 95% CI (g = −0.33 to −0.04) encompassed effects ranging from a minimal/near-null reduction to a small reduction, while only three effect sizes contributed to the pooled estimate. The limited information size therefore reduced confidence in the precision of the estimated effect. Statistical significance alone was not used to determine this downgrade.
**BMI**	**Risk of bias:** downgraded one level because of concerns regarding risk of bias in the contributing trials. **Inconsistency:** downgraded one level because heterogeneity was considerable (I^2 = 91.5%), indicating substantial variation in BMI responses across studies. **Imprecision:** no additional downgrade was applied. Although the 95% CI was relatively wide (MD = −2.91 to −0.06 kg/m²) and approached the null, it remained entirely in the direction of BMI reduction. Because no validated clinically important threshold for BMI change was prespecified for this review, the interval was not judged to cross a predefined decision threshold sufficient to warrant a separate downgrade for imprecision.
**Weight**	**Risk of bias:** downgraded one level because of concerns regarding risk of bias in the contributing trials. **Inconsistency:** no downgrade was applied because heterogeneity was moderate and the overall direction of effects was reasonably consistent across studies. **Imprecision:** no downgrade was applied because the 95% CI (MD = −4.60 to −1.51 kg) remained entirely in the direction of weight reduction and did not extend to a near-null average effect. The wider prediction interval was considered separately as reflecting between-study variability rather than imprecision of the pooled mean estimate.
**WC**	**Risk of bias:** downgraded one level because of concerns regarding risk of bias in the contributing trials. **Inconsistency:** no downgrade was applied because statistical heterogeneity was negligible (I^2 = 0%). **Imprecision:** downgraded one level because the estimate was based on a limited overall information size (four studies, five effect sizes, 202 participants). Although the 95% CI (MD = −3.59 to −1.41 cm) remained in the direction of reduction, the available information was considered insufficient to provide high confidence in the stability and precision of the pooled estimate.
**Total Fat Mass**	**Risk of bias:** downgraded one level because of concerns regarding risk of bias in the contributing trials. **Inconsistency:** no downgrade was applied because statistical heterogeneity was negligible (I^2 = 0%). **Imprecision:** downgraded one level because the pooled estimate was based on a limited overall information size (four studies, five effect sizes, 283 participants). Although the 95% CI (MD = −3.48 to −1.12 kg) remained in the direction of fat-mass reduction, the limited amount of information reduced confidence in the precision and stability of the estimated effect.

Supplementary Material SE. Summary of Subgroup Analyses

（1）E2 subgroup Analysis

Subgroup variable		P within subgroup	P for subgroup difference
Fasting status	Yes	0.0064	0.1701
	Unclear	0.7370	
Health status	Otherwise healthy, overweight	0.9341	0.7103
	Otherwise healthy, Obesity	0.0297	
	PCOS	0.0905	
	Breast cancer		
	FD		
	Infertile women		
intensity	Low	0.7956	0.1873
	Moderate	0.0059	
	high	0.0121	
Measurement method	IEMA		0.6899
	CLIA	0.6226	
	RIA	0.0125	
	UPLC–MS/MS		
	ELISA	0.7370	
	GC–MS		
	NR	0.2838	
Type	Jogging	0.5050	0.9879
	Combined aerobic	0.0037	
	Cycling	0.3367	
	Walking	0.4568	
Menopausal status	Postmenopausal	0.2059	0.7519
	Premenopausal	0.0444	
	Mixed		
	perimenopausal		

（2）SHBG subgroup analysis

Subgroup variable		P within subgroup	P for subgroup difference
Fasting status	Yes	0.0278	0.1584
	Unclear		
Health status	Otherwise healthy, overweight		0.2803
	Otherwise healthy, Obesity	0.0507	
	PCOS	0.1178	
	Breast cancer		
intensity	Low		0.7560
	Moderate	0.0061	
	high	0.2655	
**Measurement method**	CLIA	0.0081	**＜0.05**
	RIA	0.1827	
	ELISA		
	NR		
Menopausal status	Postmenopausal	0.0611	0.2789
	Premenopausal	0.1178	
	Mixed		
	perimenopausal		
**Type**	Jogging	0.4446	**＜0.05**
	Combined aerobic	0.2573	
	Cycling	0.1178	
	Walking		

Supplementary Material SF. Leave-one-out sensitivity analyses

(1)E2

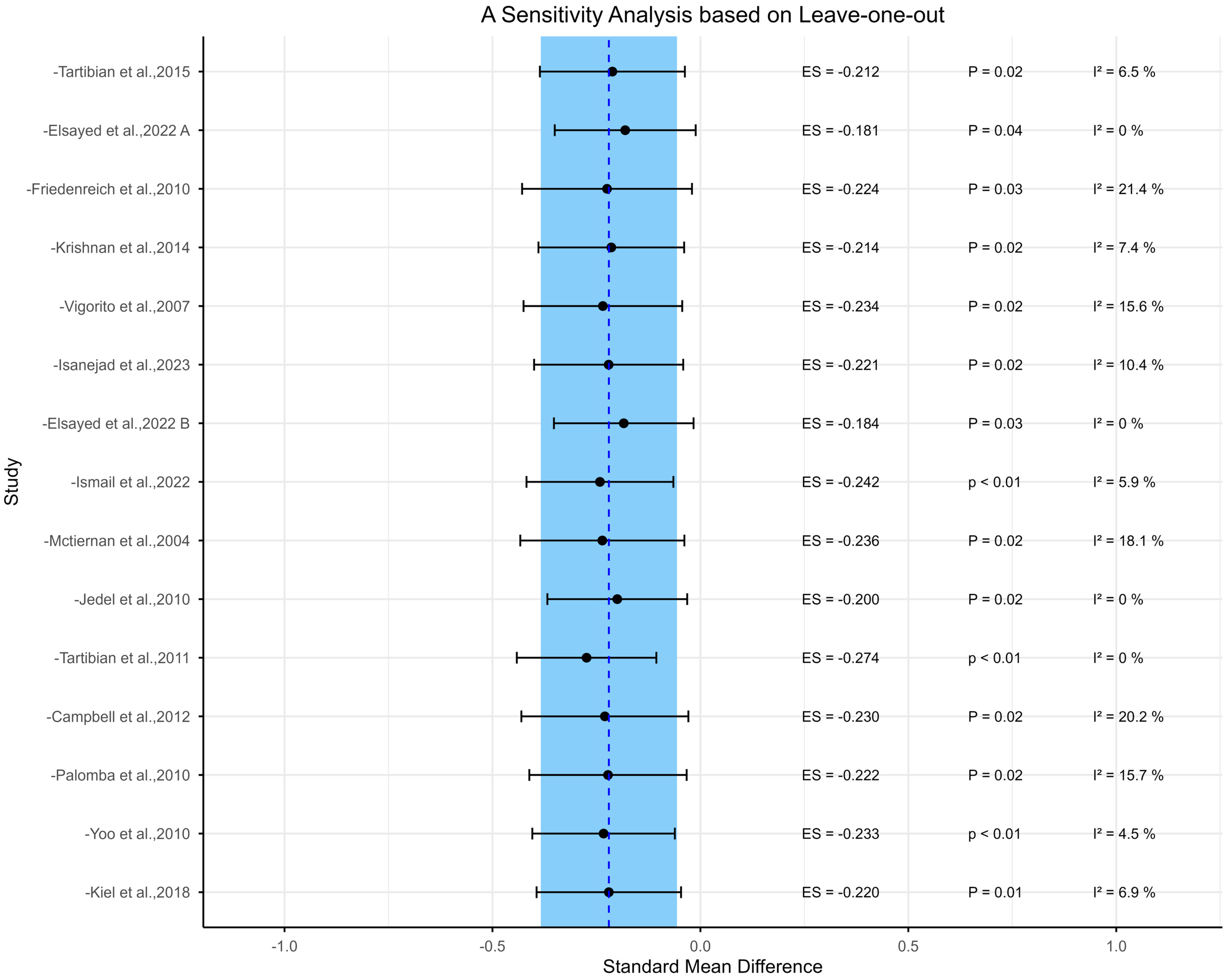

(2)E1

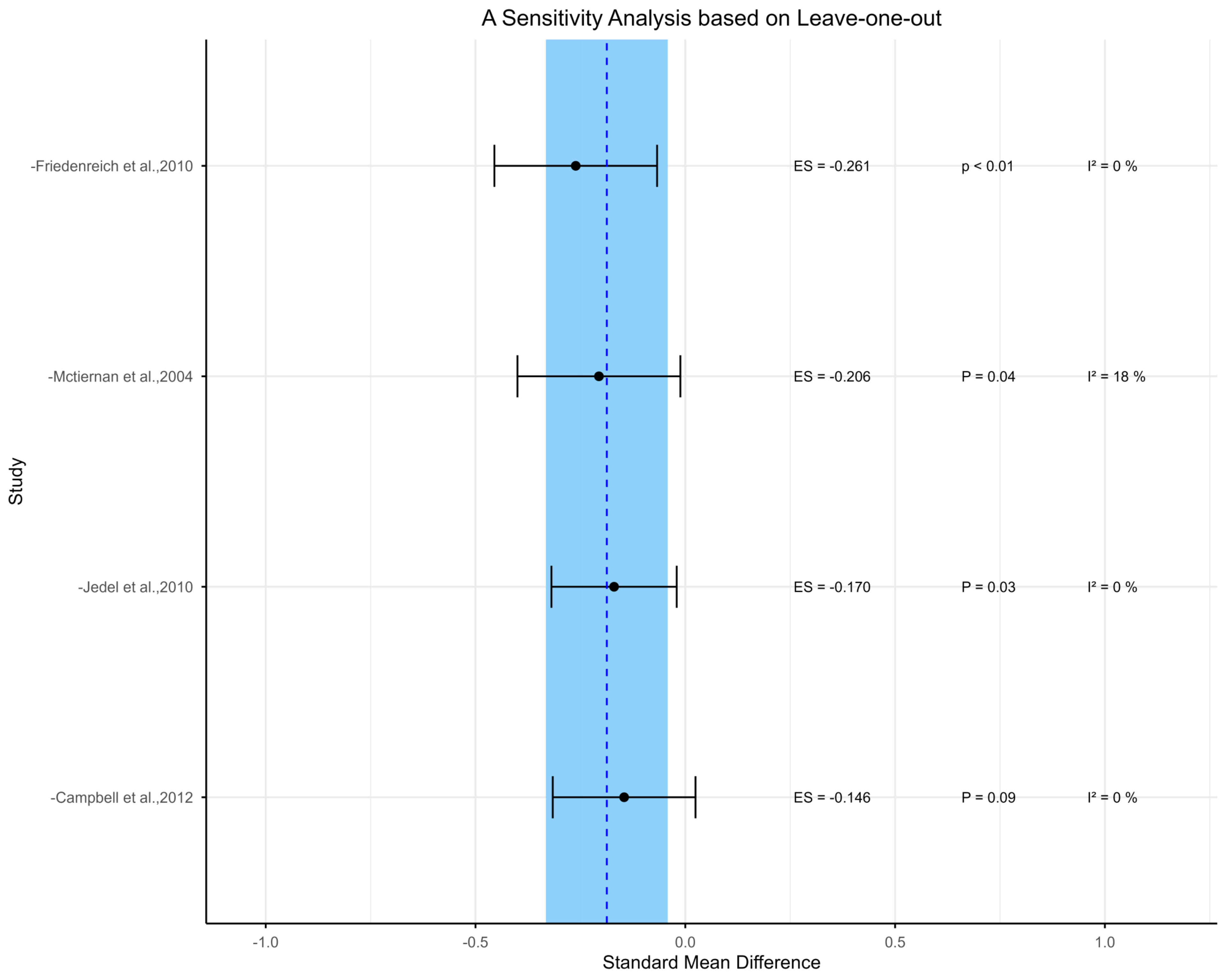

A Sensitivity Analysis based on Leave-one-out
Study
-Friedenreich et al.,2010
-Mctiernan et al.,2004
-Jedel et al.,2010
-Campbell et al.,2012
ES = -0.261
p < 0.01
I² = 0 %
ES = -0.206
P = 0.04
I² = 18 %
ES = -0.170
P = 0.03
I² = 0 %
ES = -0.146
P = 0.09
I² = 0 %
-1.0
-0.5
0.0
0.5
1.0
Standard Mean Difference

(3)SHBG

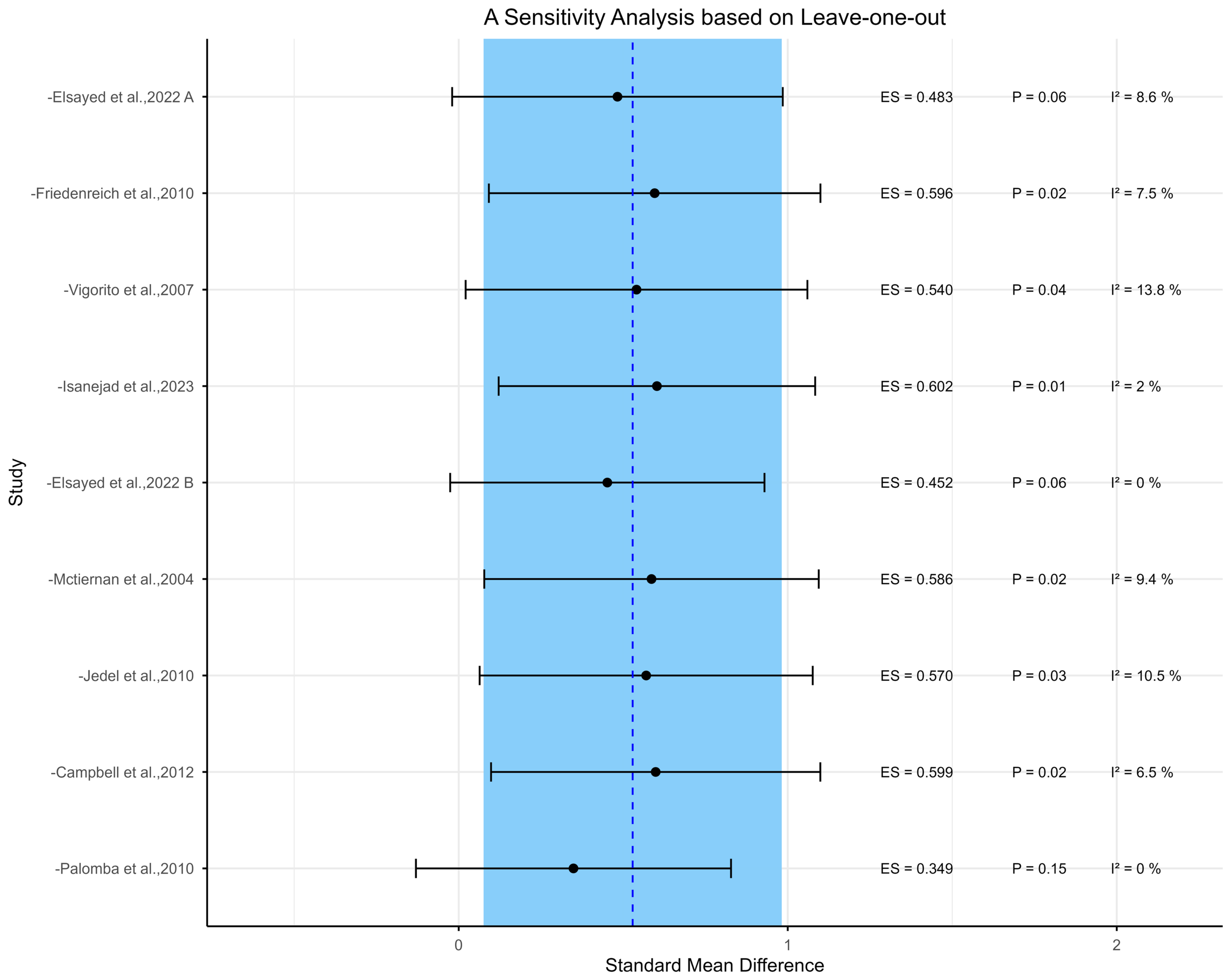

A Sensitivity Analysis based on Leave-one-out
Study
-Elsayed et al.,2022 A
-Friedenreich et al.,2010
-Vigorito et al.,2007
-Isanejad et al.,2023
-Elsayed et al.,2022 B
-Mctiernan et al.,2004
-Jedel et al.,2010
-Campbell et al.,2012
-Palomba et al.,2010
ES = 0.483 P = 0.06 I² = 8.6 %
ES = 0.596 P = 0.02 I² = 7.5 %
ES = 0.540 P = 0.04 I² = 13.8 %
ES = 0.602 P = 0.01 I² = 2 %
ES = 0.452 P = 0.06 I² = 0 %
ES = 0.586 P = 0.02 I² = 9.4 %
ES = 0.570 P = 0.03 I² = 10.5 %
ES = 0.599 P = 0.02 I² = 6.5 %
ES = 0.349 P = 0.15 I² = 0 %
0
1
2
Standard Mean Difference

(4) Free estradiol

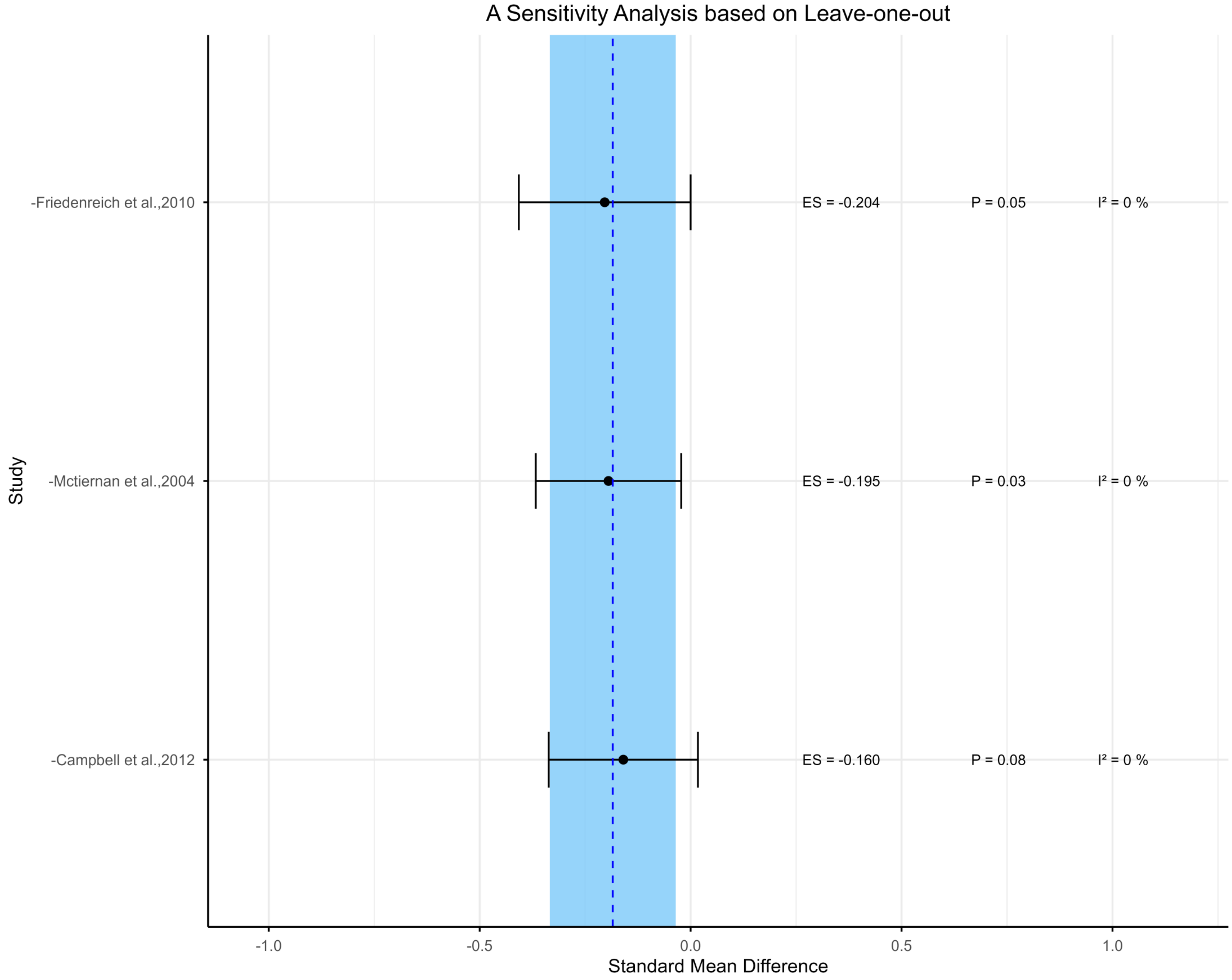

(5)BMI

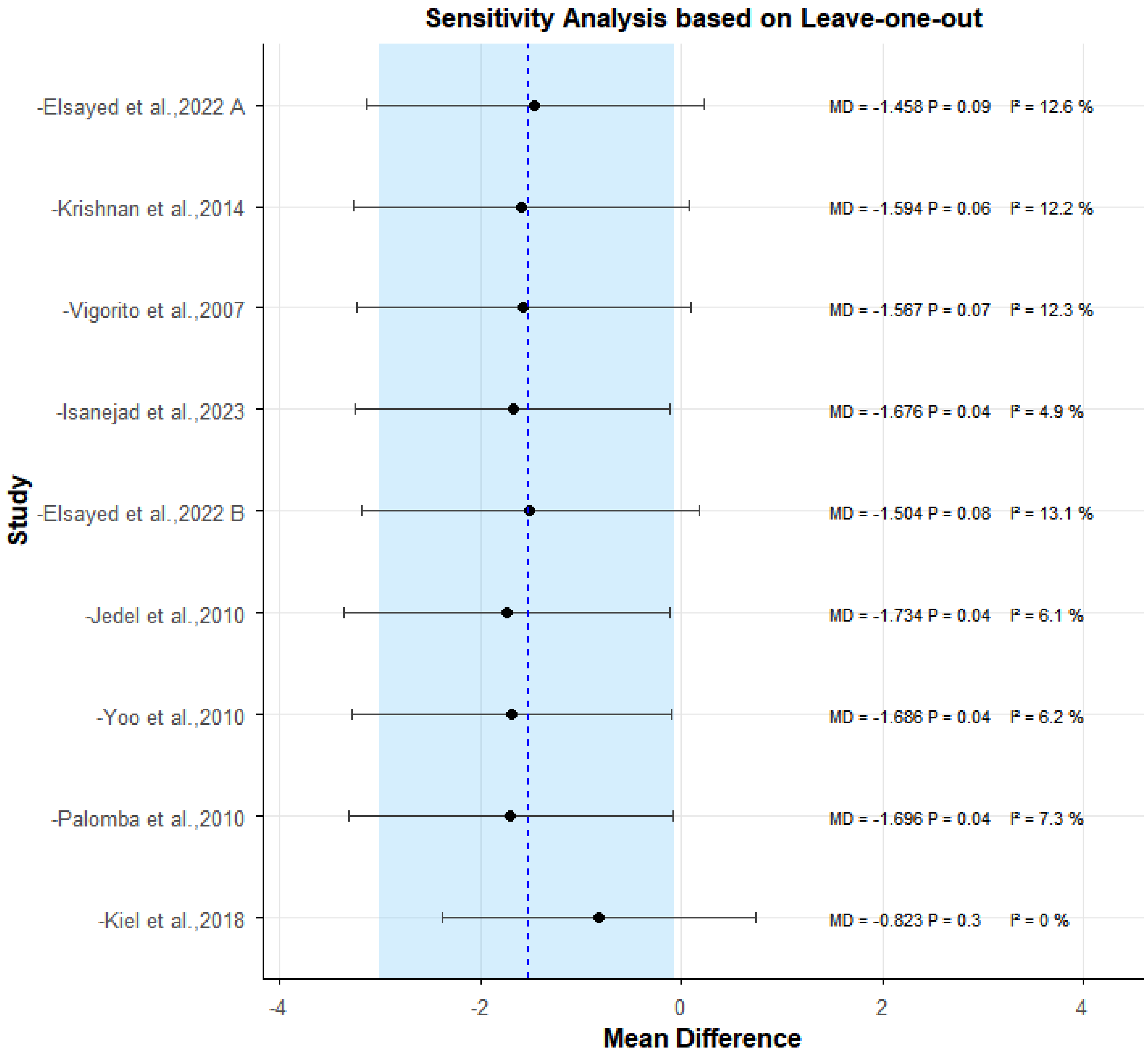

(6)Weight

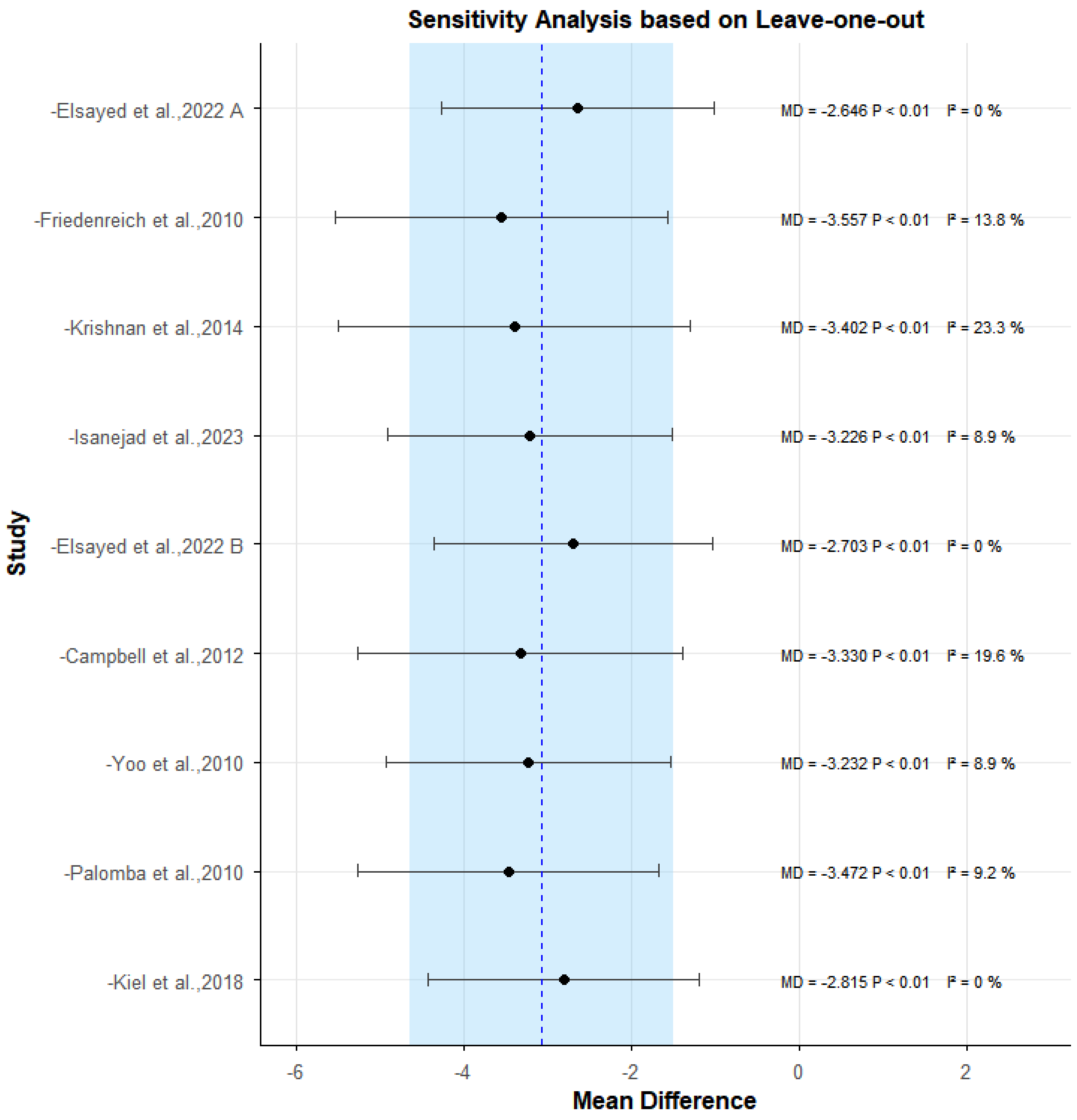
Sensitivity Analysis based on Leave-one-out
-Elsayed et al.,2022 A
-Friedenreich et al.,2010
-Krishnan et al.,2014
-Isanejad et al.,2023
-Elsayed et al.,2022 B
-Campbell et al.,2012
-Yoo et al.,2010
-Palomba et al.,2010
-Kiel et al.,2018
Study
MD = -2.646 P < 0.01 I² = 0 %
MD = -3.557 P < 0.01 I² = 13.8 %
MD = -3.402 P < 0.01 I² = 23.3 %
MD = -3.226 P < 0.01 I² = 8.9 %
MD = -2.703 P < 0.01 I² = 0 %
MD = -3.330 P < 0.01 I² = 19.6 %
MD = -3.232 P < 0.01 I² = 8.9 %
MD = -3.472 P < 0.01 I² = 9.2 %
MD = -2.815 P < 0.01 I² = 0 %
-6
-4
-2
0
2
Mean Difference

(7)WC

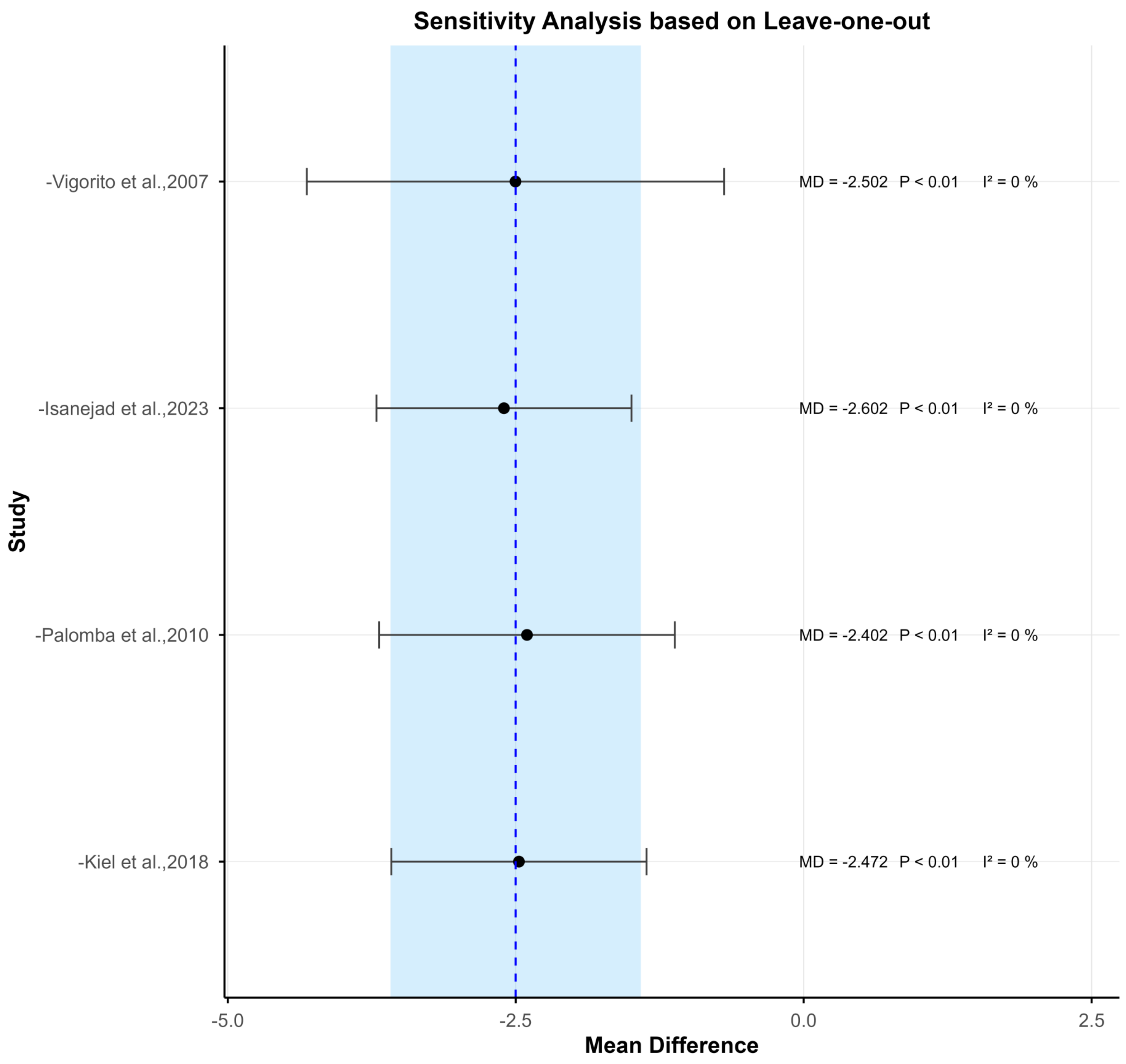

Sensitivity Analysis based on Leave-one-out
-Vigorito et al.,2007
-Isanejad et al.,2023
-Palomba et al.,2010
-Kiel et al.,2018
MD = -2.502 P < 0.01 I² = 0 %
MD = -2.602 P < 0.01 I² = 0 %
MD = -2.402 P < 0.01 I² = 0 %
MD = -2.472 P < 0.01 I² = 0 %
Study
-5.0
-2.5
0.0
2.5
Mean Difference

(8)TFM

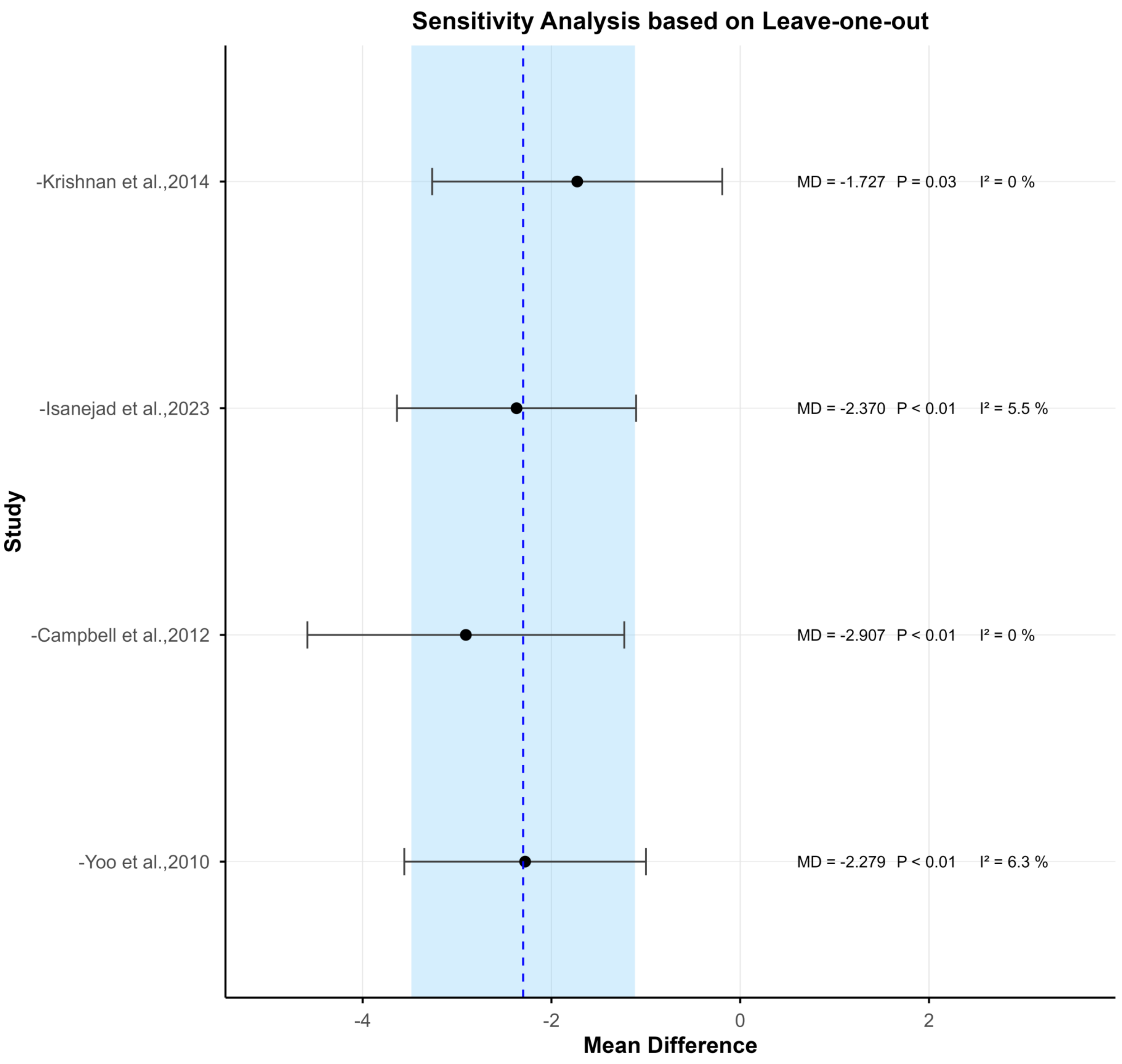

Sensitivity Analysis based on Leave-one-out
-Krishnan et al.,2014
MD = -1.727 P = 0.03 I² = 0 %
-Isanejad et al.,2023
MD = -2.370 P < 0.01 I² = 5.5 %
-Campbell et al.,2012
MD = -2.907 P < 0.01 I² = 0 %
-Yoo et al.,2010
MD = -2.279 P < 0.01 I² = 6.3 %
Study
-4
-2
0
2
Mean Difference

Supplementary Material SG. Influence Diagnostics Using Cook's Distance

（1）E2

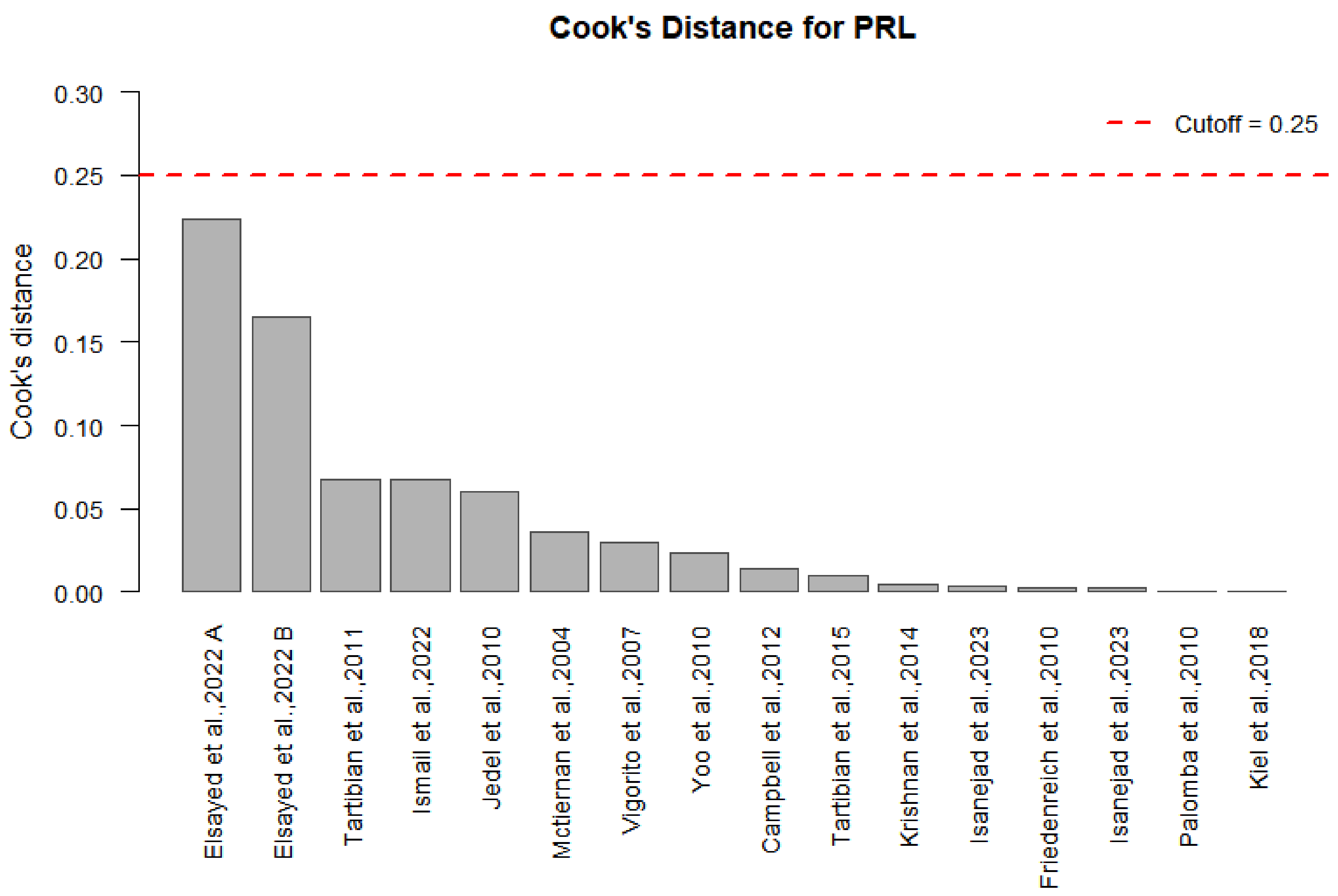

（2）E1

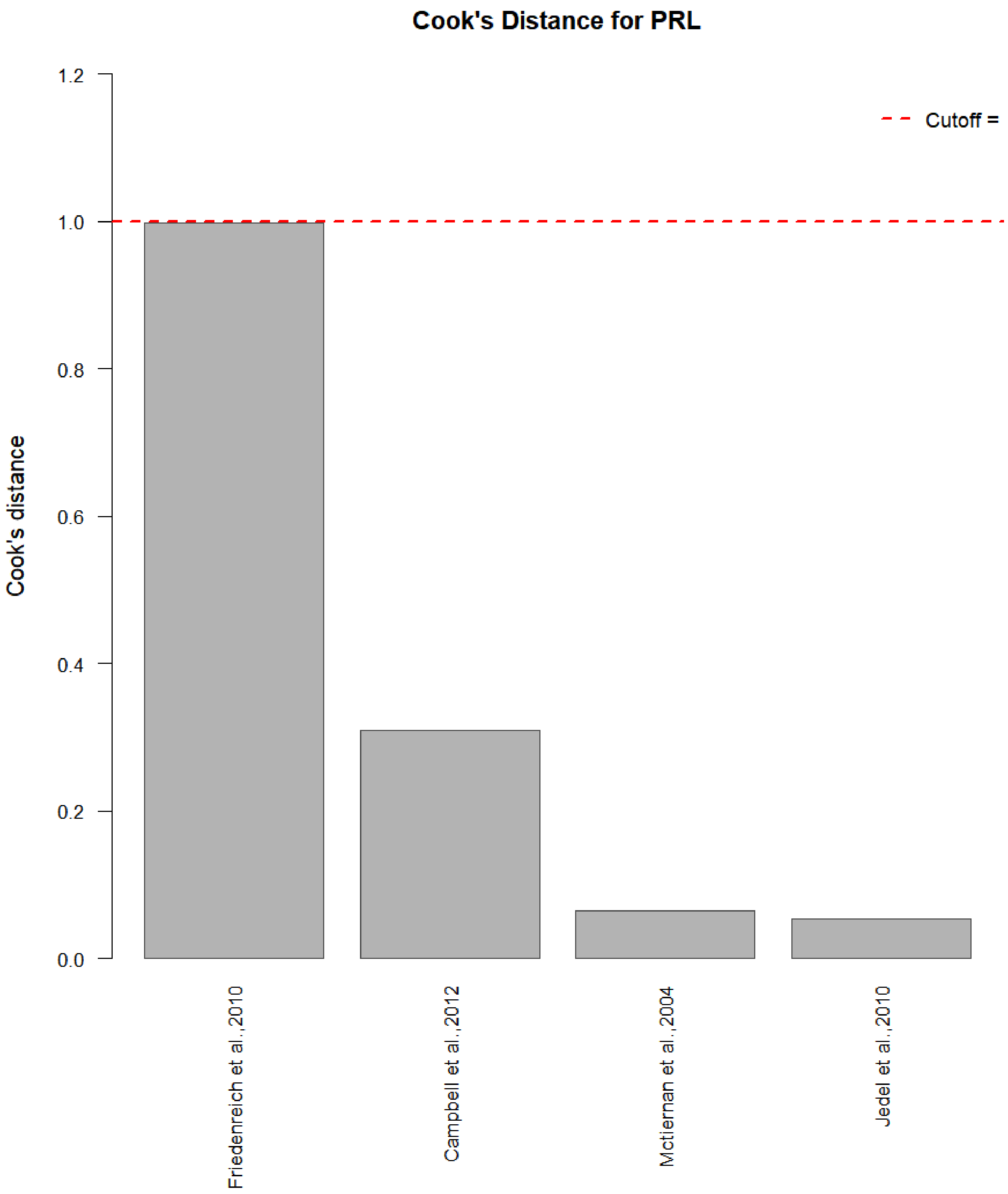
Cook's Distance for PRL
Cook's distance
1.2
1.0
0.8
0.6
0.4
0.2
0.0
Cutoff =
Friedenreich et al.,2010
Campbell et al.,2012
Mctiernan et al.,2004
Jedel et al.,2010

（3）SHBG

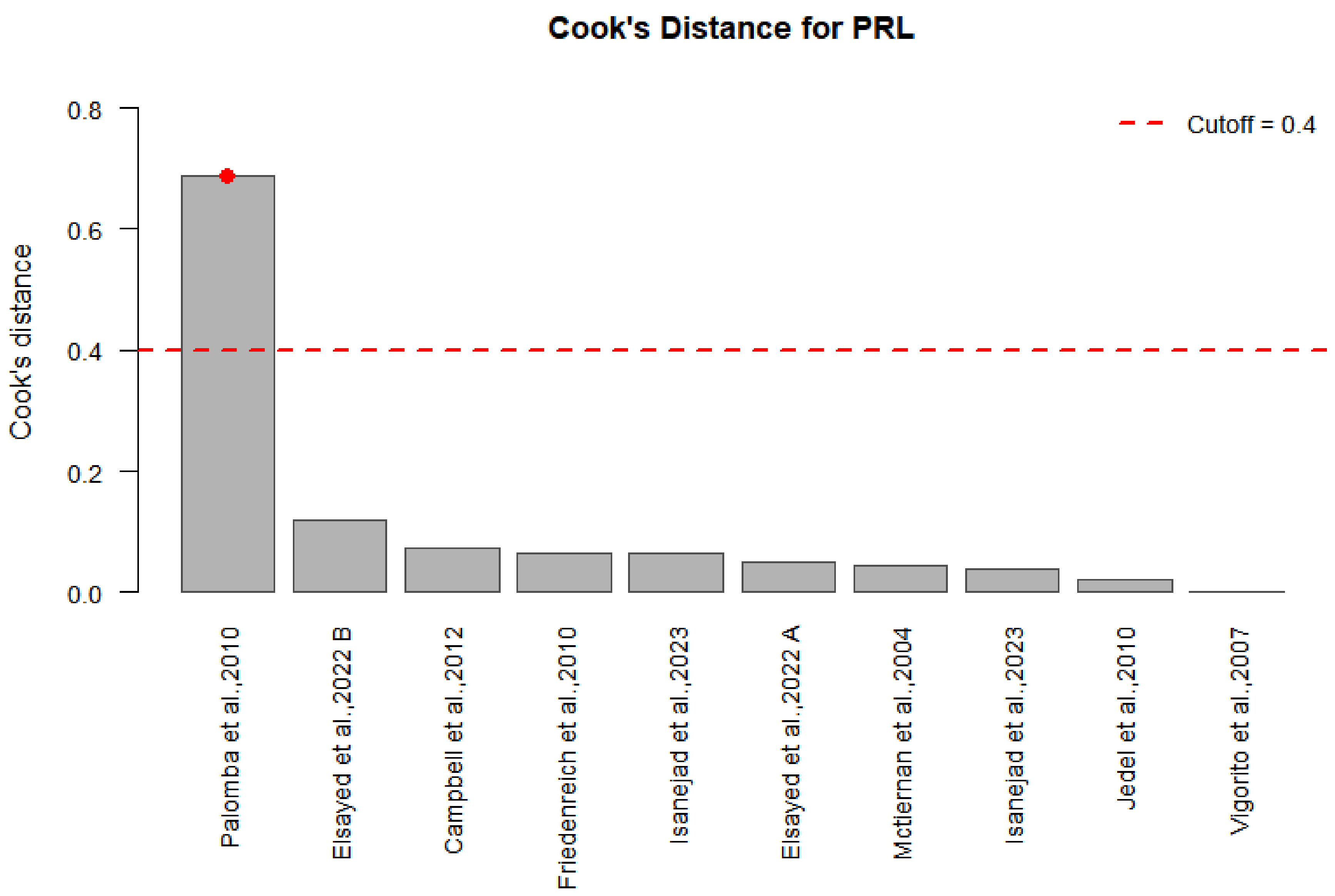

Cook's Distance for PRL
Cook's distance
0.0
0.2
0.4
0.6
0.8
Cutoff = 0.4
Palomba et al.,2010
Elsayed et al.,2022 B
Campbell et al.,2012
Friedenreich et al.,2010
Isanejad et al.,2023
Elsayed et al.,2022 A
Mctiernan et al.,2004
Isanejad et al.,2023
Jedel et al.,2010
Vigorito et al.,2007

(4) TFM

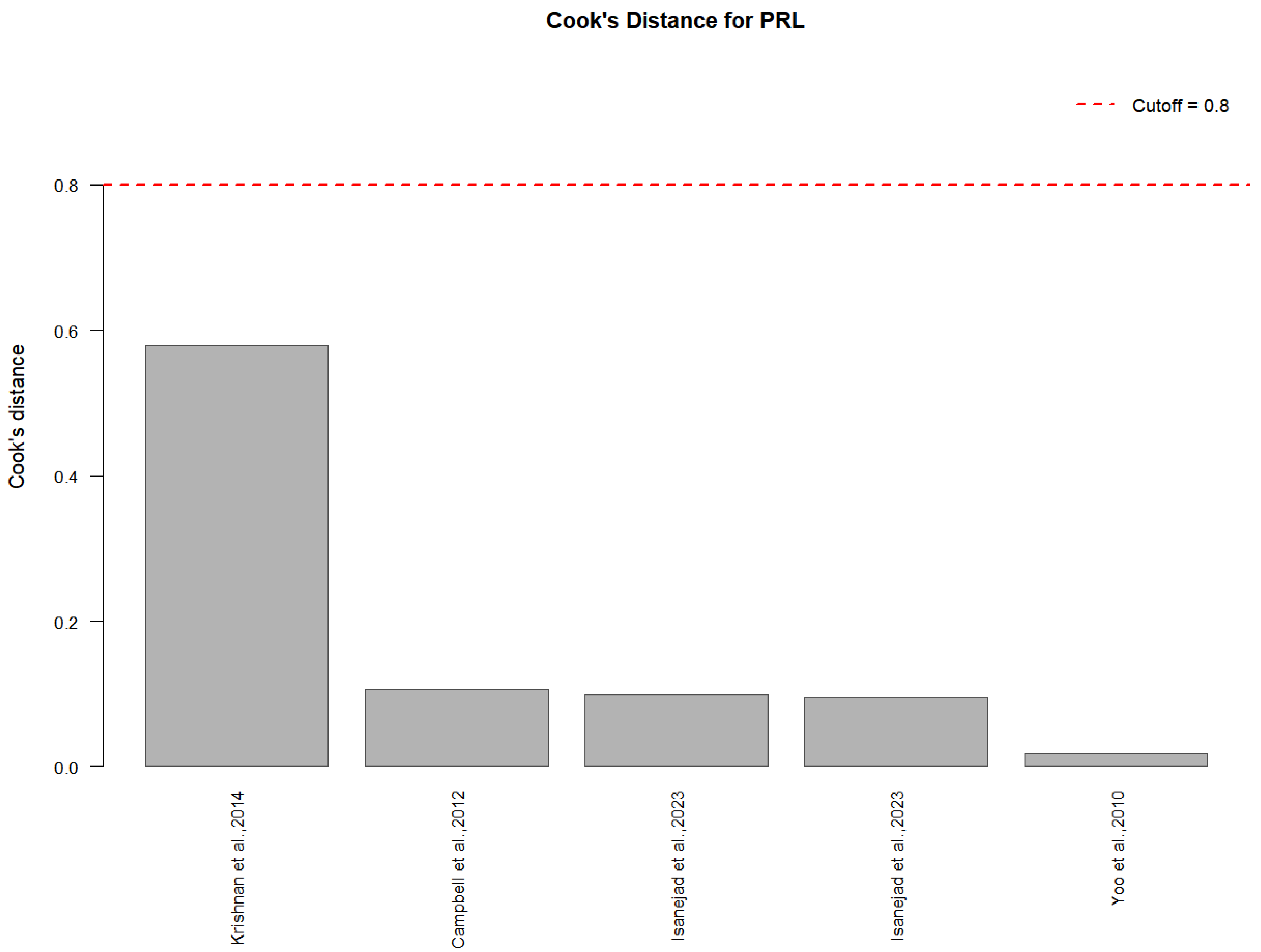

Cook's Distance for PRL
Cutoff = 0.8
Cook's distance
0.0
0.2
0.4
0.6
0.8
Krishnan et al.,2014
Campbell et al.,2012
Isanejad et al.,2023
Isanejad et al.,2023
Yoo et al.,2010

Supplementary Material SH. Sensitivity Analyses Using Alternative Pre－Post Correlation Coefficients

（1）E2

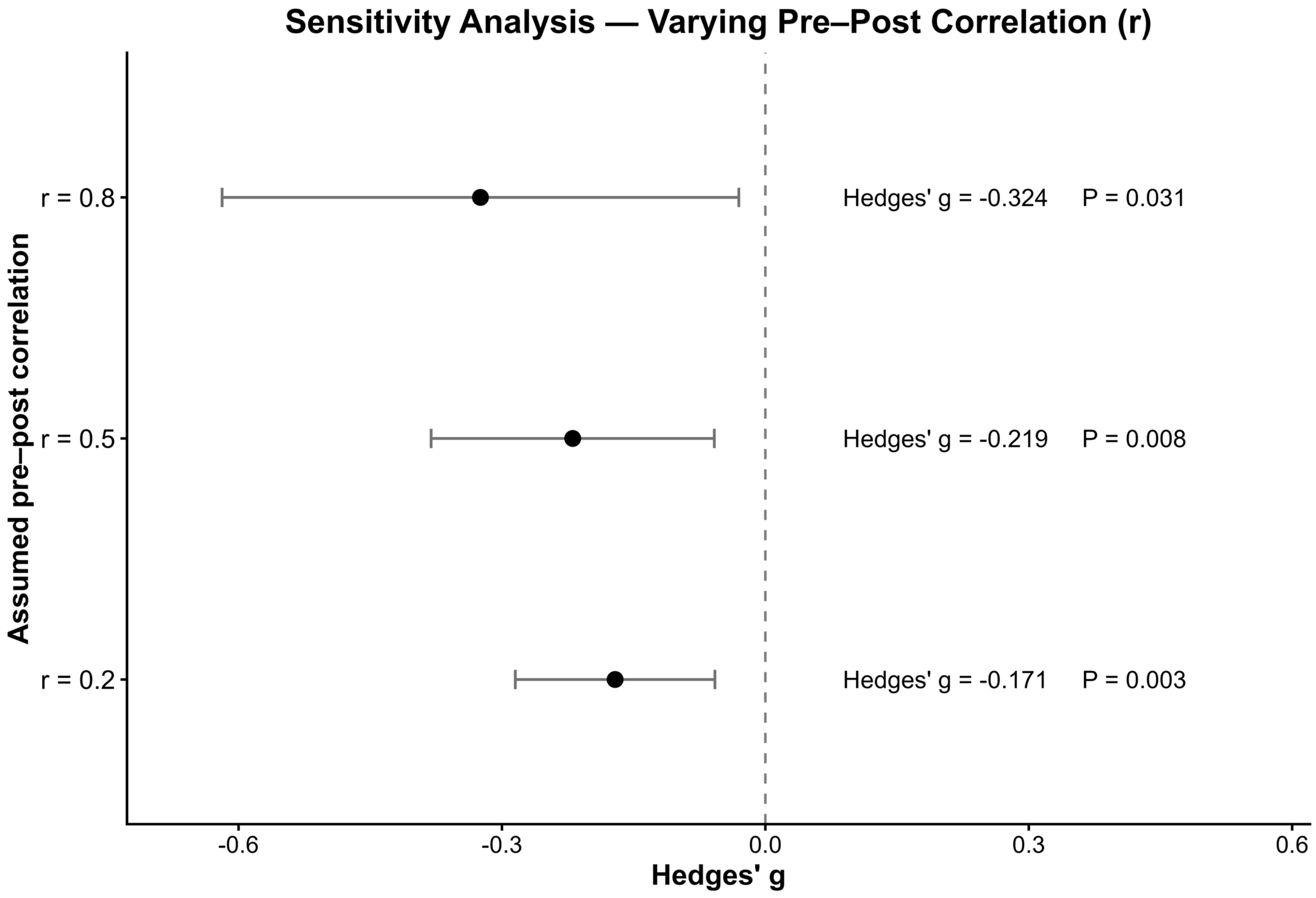

（2）E1

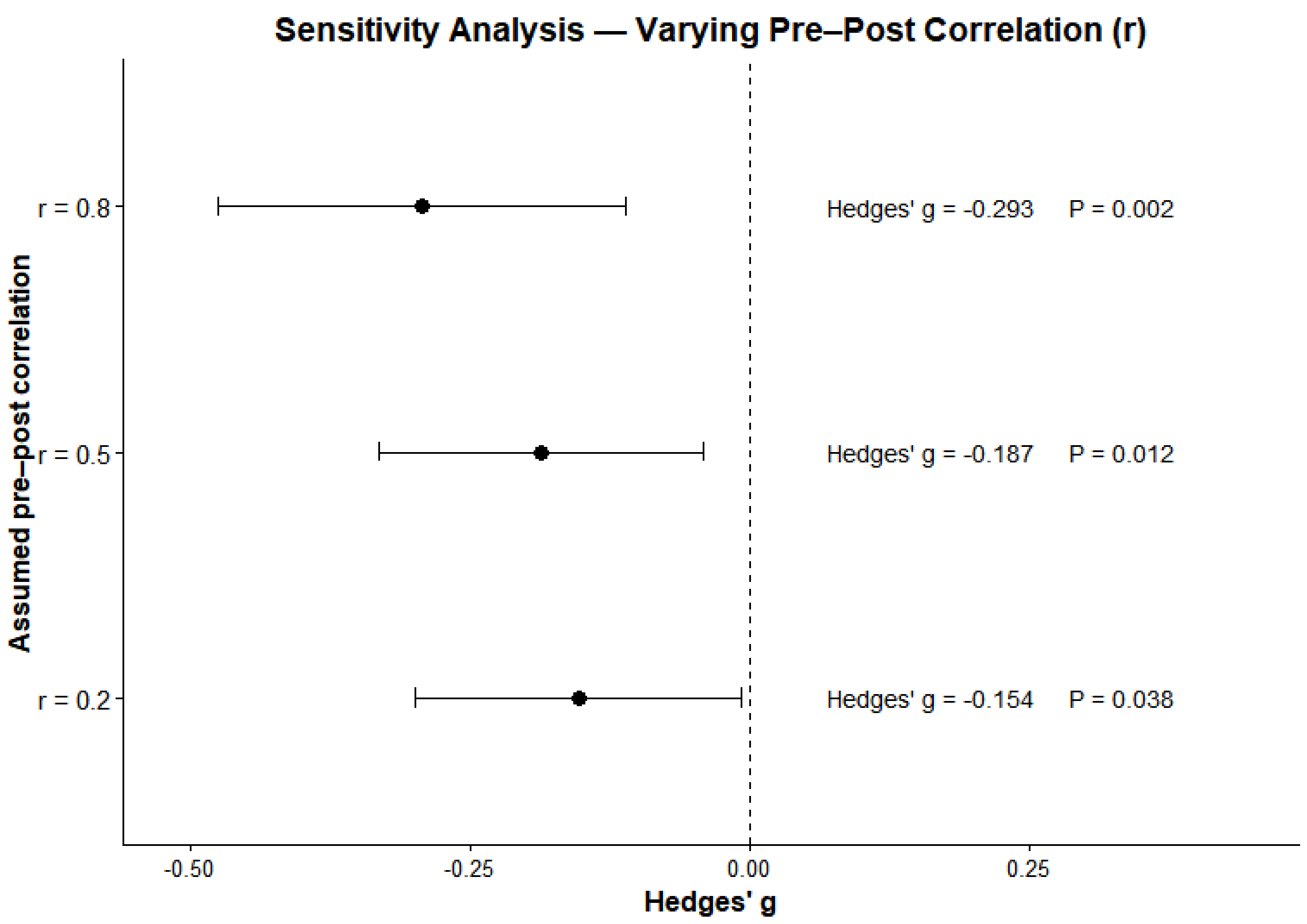
Sensitivity Analysis — Varying Pre–Post Correlation (r)
Assumed pre–post correlation
r = 0.8
r = 0.5
r = 0.2
Hedges' g = -0.293 P = 0.002
Hedges' g = -0.187 P = 0.012
Hedges' g = -0.154 P = 0.038
-0.50
-0.25
0.00
0.25
Hedges' g

（3）SHBG

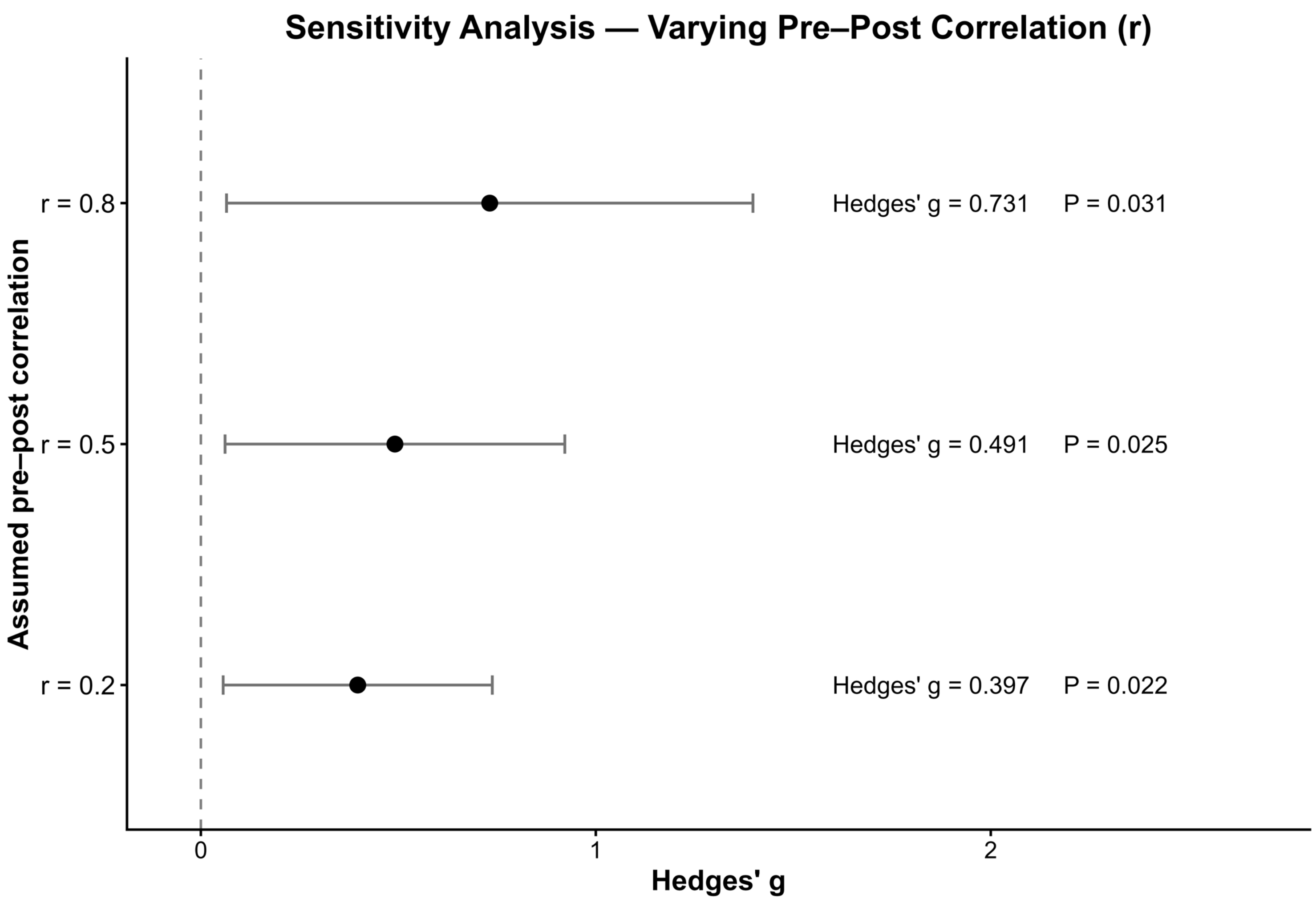

Sensitivity Analysis — Varying Pre–Post Correlation (r)
Assumed pre–post correlation
r = 0.8
r = 0.5
r = 0.2
Hedges' g = 0.731 P = 0.031
Hedges' g = 0.491 P = 0.025
Hedges' g = 0.397 P = 0.022
0
1
2
Hedges' g

（4）Free E2

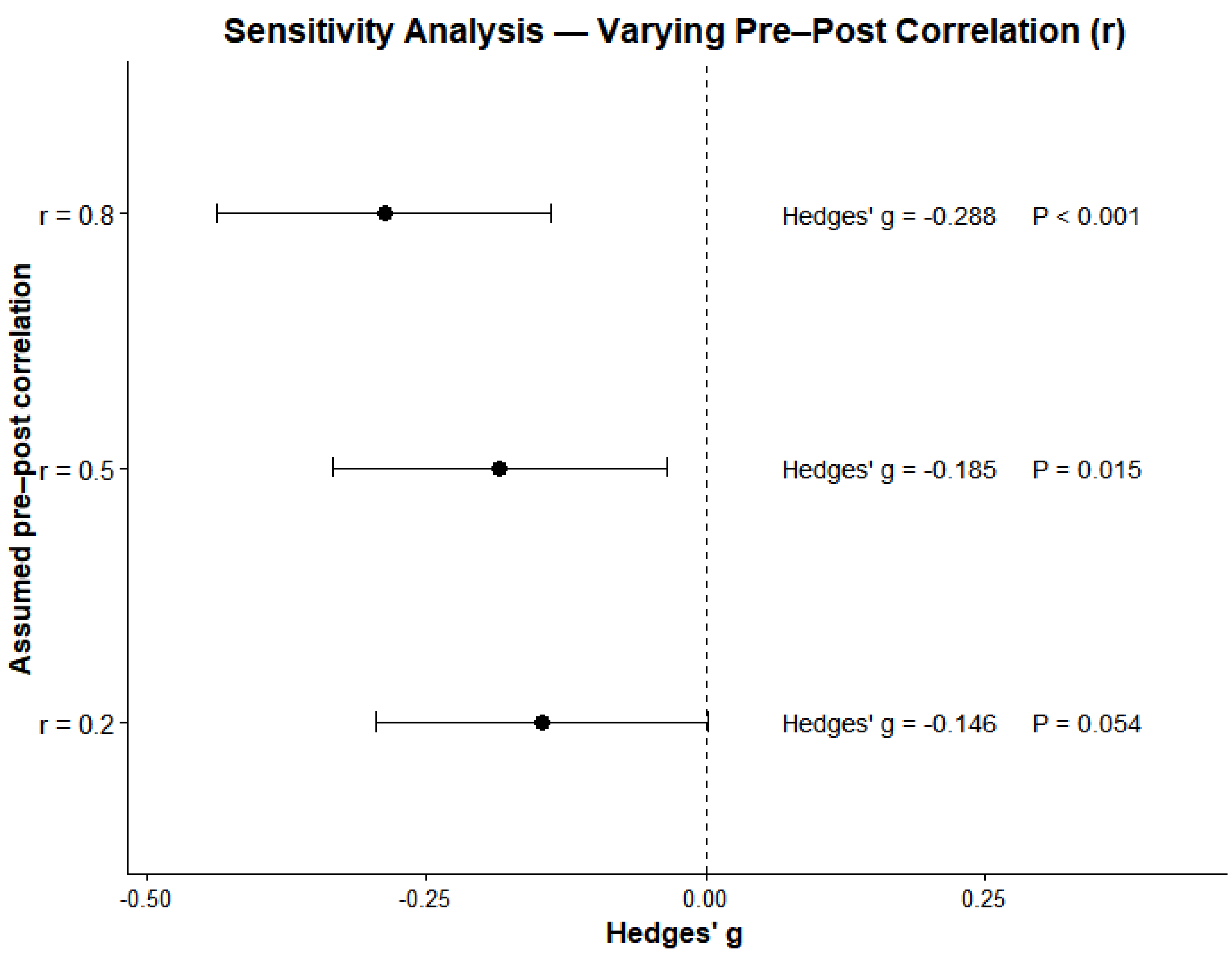
Sensitivity Analysis — Varying Pre–Post Correlation (r)
Assumed pre–post correlation
r = 0.8
r = 0.5
r = 0.2
Hedges' g = -0.288
P < 0.001
Hedges' g = -0.185
P = 0.015
Hedges' g = -0.146
P = 0.054
-0.50
-0.25
0.00
0.25
Hedges' g

（5）BMI

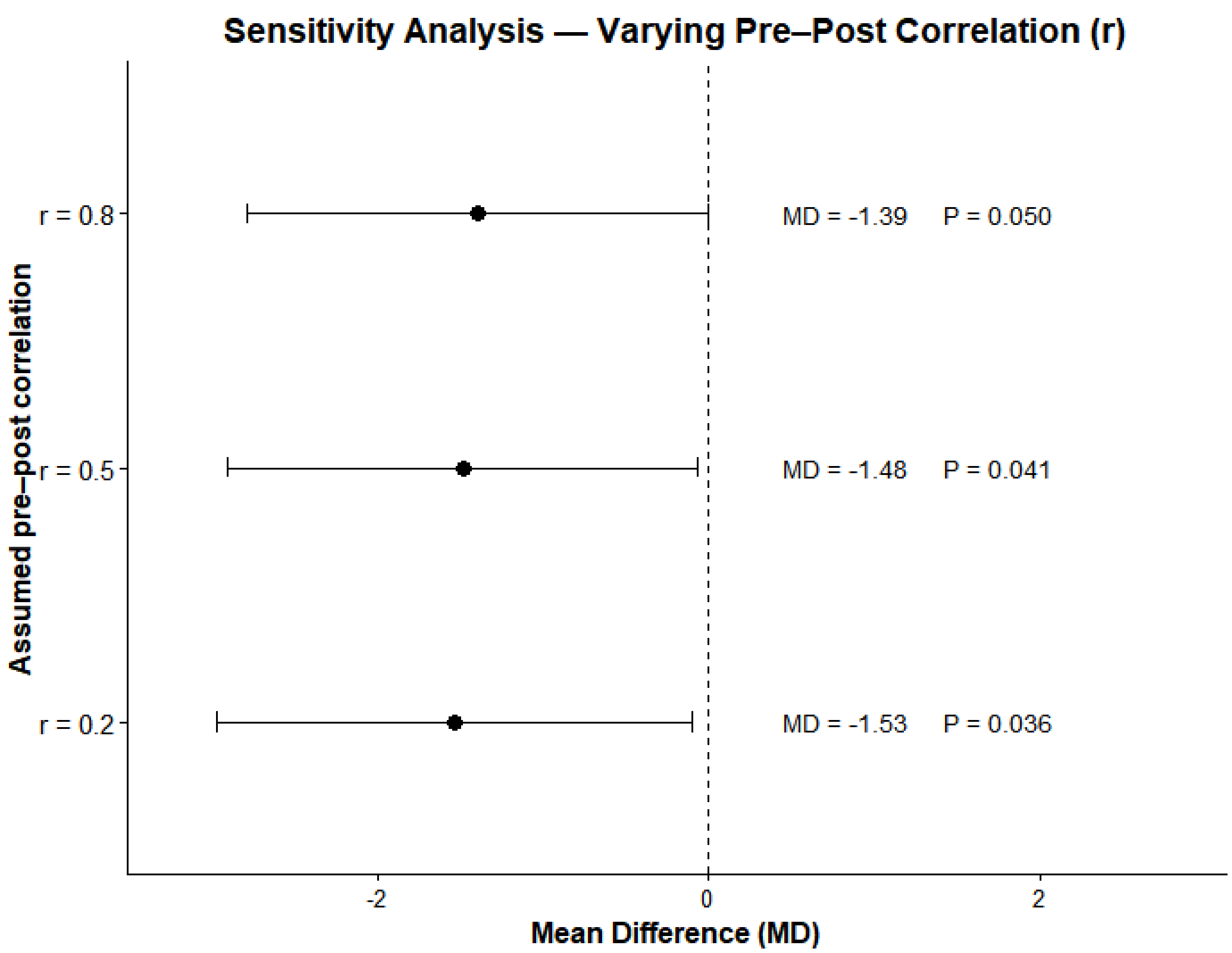
Sensitivity Analysis — Varying Pre–Post Correlation (r)
Assumed pre–post correlation
r = 0.8
r = 0.5
r = 0.2
MD = -1.39 P = 0.050
MD = -1.48 P = 0.041
MD = -1.53 P = 0.036
-2
0
2
Mean Difference (MD)

(6) Weight

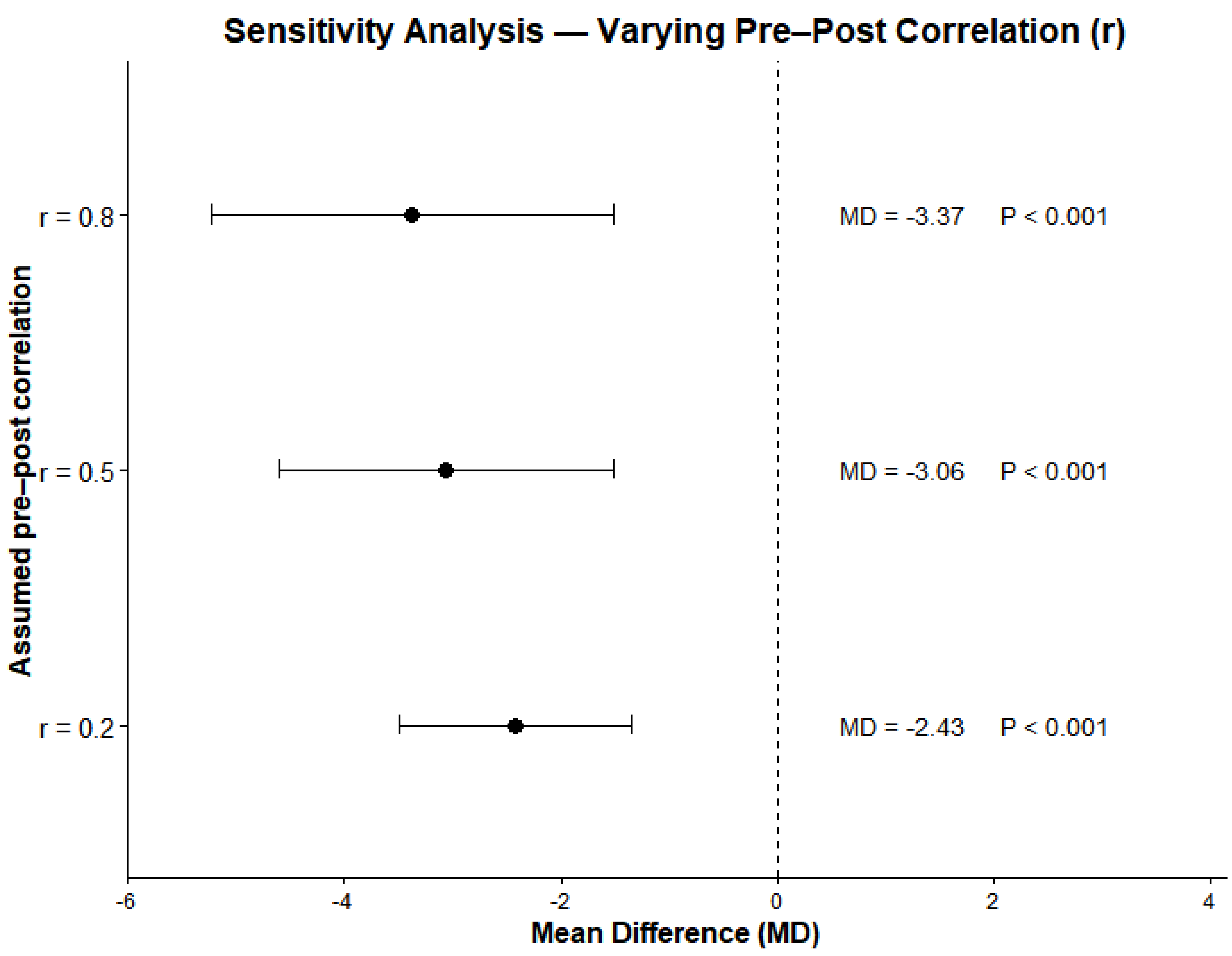
Sensitivity Analysis — Varying Pre–Post Correlation (r)
Assumed pre–post correlation
r = 0.8
r = 0.5
r = 0.2
MD = -3.37 P < 0.001
MD = -3.06 P < 0.001
MD = -2.43 P < 0.001
-6
-4
-2
0
2
4
Mean Difference (MD)

（7）WC

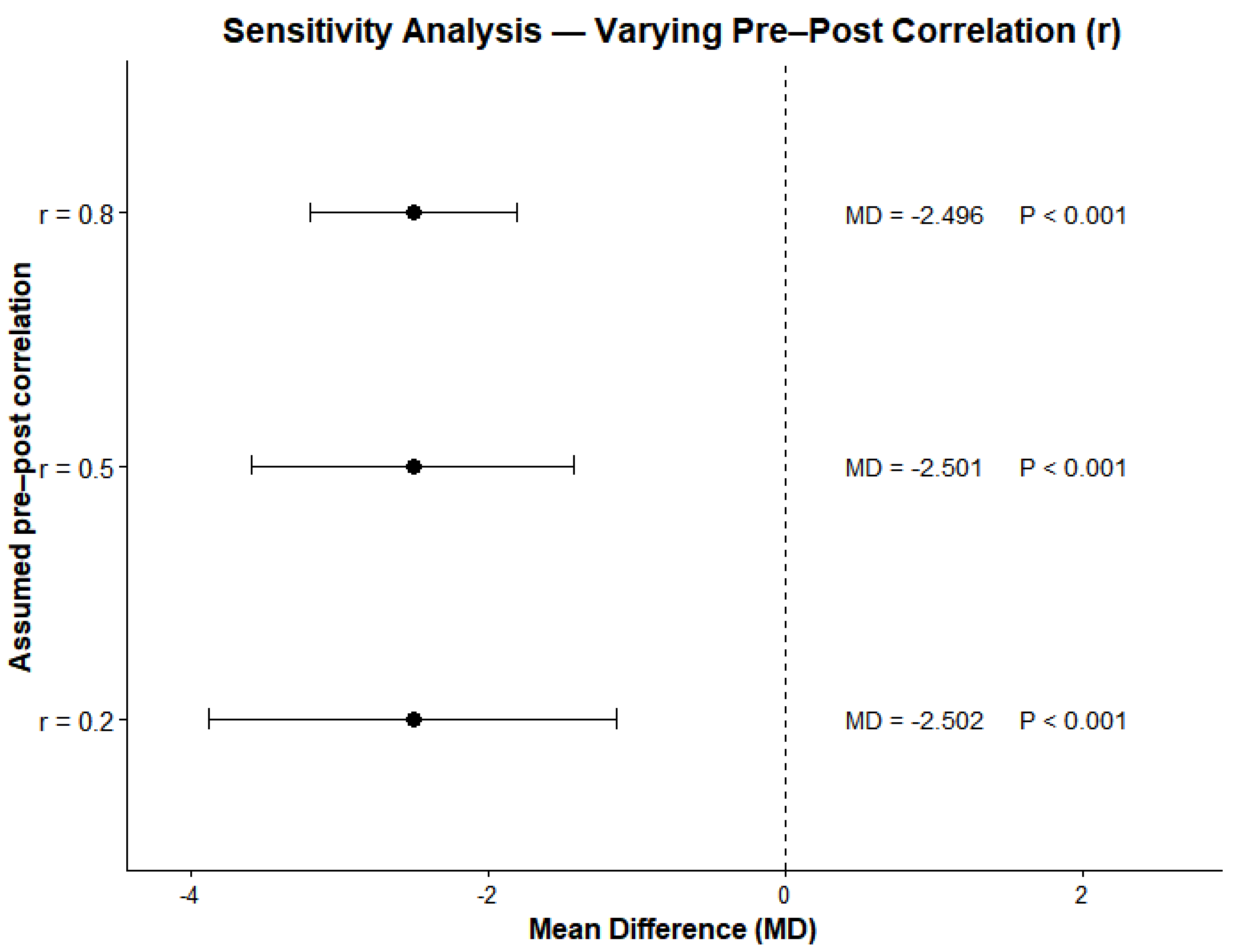
Sensitivity Analysis — Varying Pre–Post Correlation (r)
Assumed pre–post correlation
r = 0.8
r = 0.5
r = 0.2
MD = -2.496
P < 0.001
MD = -2.501
P < 0.001
MD = -2.502
P < 0.001
-4
-2
0
2
Mean Difference (MD)

（8）TFM

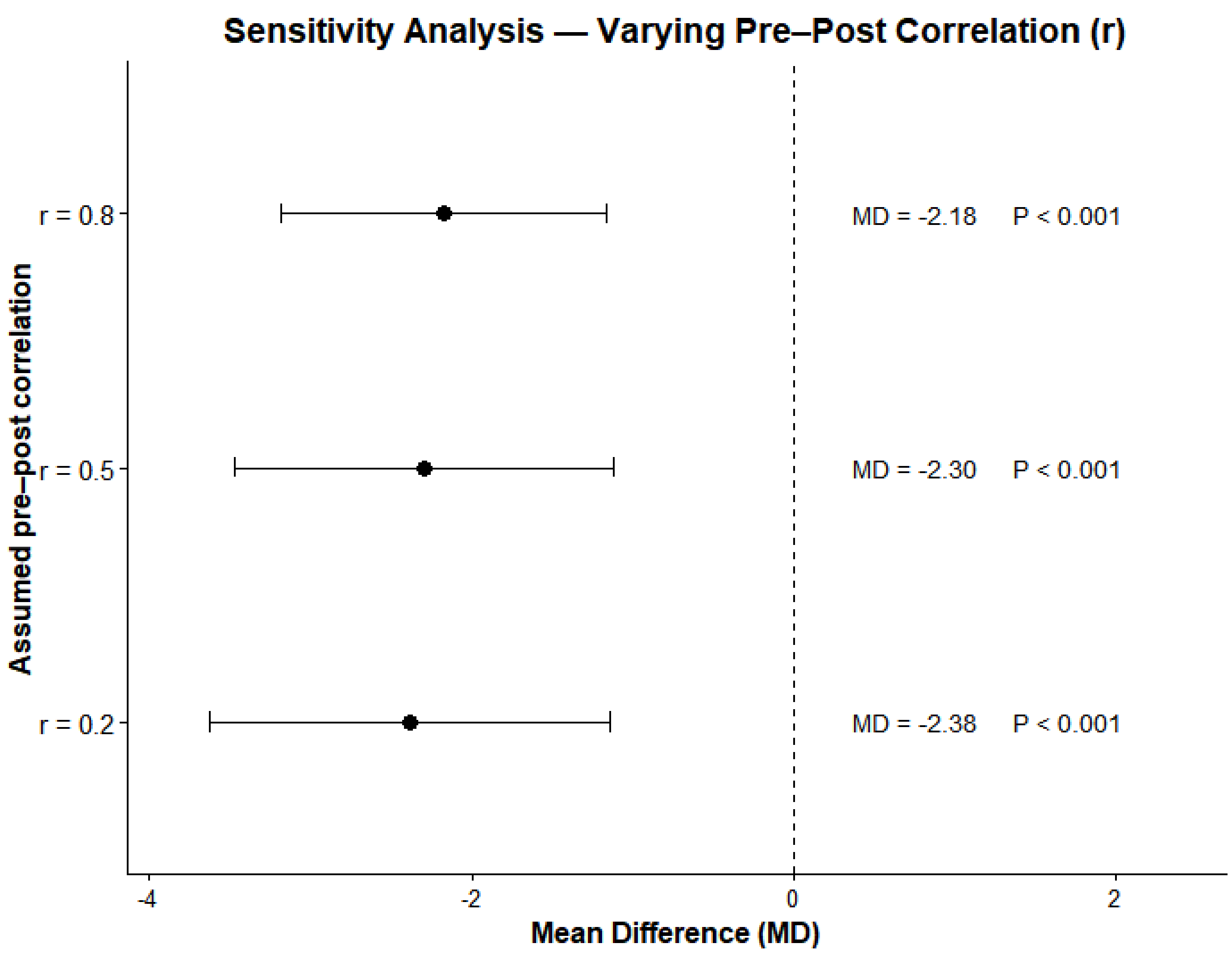

Supplementary Material SI. Sensitivity Analyses Excluding Studies at High Risk of Bias

Outcome	SMD	95% CI	P value	I^2
E2 (excluding high-risk studies)	-0.17	-0.29 to -0.05	$P < 0.05$	34.7%
E1 (excluding high-risk studies)	-0.16	−0.31 to −0.01	$P < 0.05$	0%
SHBG (excluding high-risk studies)	0.52	0.04 to 1.00	$P < 0.05$	85.8%
**Outcome**	**MD**	**95% CI**	**P value**	**I^2**
Total fat mass (excluding high-risk studies)	−1.34	−2.58 to −0.10	$P < 0.05$	0%
Weight (excluding high-risk studies)	−2.62	−4.11 to −1.13	$P < 0.05$	39.8%
BMI (excluding high-risk studies)	−0.94	−1.94 to 0.06	$P = 0.065$	87.6%
